# Modeling the Shape of Synaptic Spines by Their Actin Dynamics

**DOI:** 10.3389/fnsyn.2020.00009

**Published:** 2020-03-10

**Authors:** Mayte Bonilla-Quintana, Florentin Wörgötter, Christian Tetzlaff, Michael Fauth

**Affiliations:** ^1^Department for Computational Neuroscience, Third Institute of Physics-Biophysics, Georg-August-University, Göttingen, Germany; ^2^Bernstein Center for Computational Neuroscience, Georg-August-University, Göttingen, Germany

**Keywords:** dendritic spines, actin, simulations, model, spontaneous shape change

## Abstract

Dendritic spines are the morphological basis of excitatory synapses in the cortex and their size and shape correlates with functional synaptic properties. Recent experiments show that spines exhibit large shape fluctuations that are not related to activity-dependent plasticity but nonetheless might influence memory storage at their synapses. To investigate the determinants of such spontaneous fluctuations, we propose a mathematical model for the dynamics of the spine shape and analyze it in 2D—related to experimental microscopic imagery—and in 3D. We show that the spine shape is governed by a local imbalance between membrane tension and the expansive force from actin bundles that originates from discrete actin polymerization foci. Experiments have shown that only few such polymerization foci co-exist at any time in a spine, each having limited life time. The model shows that the momentarily existing set of such foci pushes the membrane along certain directions until foci are replaced and other directions may now be affected. We explore these relations in depth and use our model to predict shape and temporal characteristics of spines from the different biophysical parameters involved in actin polymerization. Approximating the model by a single recursive equation we finally demonstrate that the temporal evolution of the number of active foci is sufficient to predict the size of the model-spines. Thus, our model provides the first platform to study the relation between molecular and morphological properties of the spine with a high degree of biophysical detail.

## 1. Introduction

Dendritic spines are small protrusions from neural dendrites, which form the post-synaptic part of most excitatory synapses in the cortex (Yuste, 2010). One of the central paradigms of neuroscience is that synapses store memories by changing their transmission efficacies during learning (Martin et al., 2000) and it has been shown that synaptic transmission efficacy correlates with size and shape of the spines. This has been mostly studied using the volume of the spine head (Matsuzaki et al., 2001, 2004; Zhou et al., 2004; Hotulainen and Hoogenraad, 2010, more details in Fauth and Tetzlaff, 2016) providing evidence for a link between spine-morphological and synaptic-functional properties. However, it recently became clear that most of the dynamic properties of changing spine volumes emerge from spontaneous spine specific processes that are not determined by the activity of the pre- or post-synaptic neuron (Dunaevsky et al., 1999; Yasumatsu et al., 2008; Dvorkin and Ziv, 2016). As such spontaneous fluctuations could affect memory functions due to the above described link (Mongillo et al., 2017), a thorough understanding of their characteristics and underlying processes is necessary. Experiments imaging the shape of dendritic spines can provide snapshots at distinct time points, but mathematical models are needed to bridge between these time points and to understand shape fluctuations and their properties. However, so far only phenomenological models have been proposed (Yasumatsu et al., 2008; Loewenstein et al., 2011; Statman et al., 2014; Hartmann et al., 2015) that describe fluctuations coarsely on a timescale of days. Here, we take a different approach by modeling the fast actin dynamics underlying shape fluctuations. This approach also allows us to explore the influence of the molecular and mechanical processes involved and to make predictions on the fluctuations when their properties vary.

**Figure 1:**
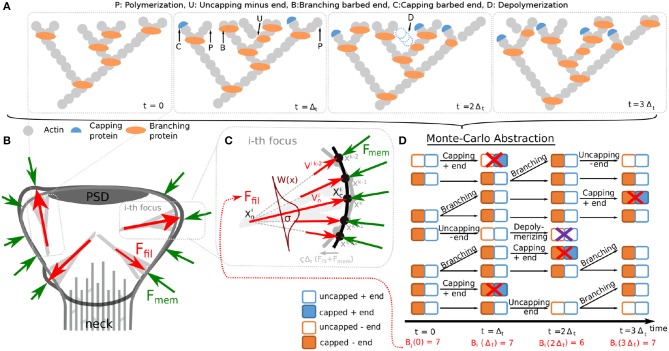
Components of the proposed spine fluctuation model. **(A)** Schematic picture of actin filaments in a polymerization foci at successive time-points. Additional to polymerization of new actin monomers at the barbed ends, other events can occur in actin filaments, such as: branching the barbed ends by inserting branching protein Arp2/3, capping barbed ends with capping proteins, uncapping minus end and depolymerizing uncapped minus ends. These events are indicated by an arrow and the corresponding first letter (see glossary above the panels). **(B)** Our model for spine fluctuation assumes that the shape of the membrane is determined by the membrane forces **F**_*mem*_ resisting bending and stretching and the forces generated by actin polymerization **F**_*fil*_ at a few foci. **(C)** Actin filaments at the foci are considered to extent laterally to the membrane. Hence, force is proportional to the number of barbed ends at the focus and attenuated by a spatial kernel *W*(*x*). The membrane is simulated by a discrete mesh (here depicted by dots) that moves every time-step proportional to imbalance of the acting forces (black membrane → gray membrane). **(D)** The dynamics of actin in each focus are abstracted to a Monte Carlo model describing the state of the barbed and pointed ends of any filament. We depict these state representations for the time course shown in **(A)**. During simulations, the transitions between different states happen according to the processes described in **(A)** with defined rates. See main text for details.

The spine shape is determined by its cytoskeleton, the main component of which is actin. Actin is a globular protein (G-actin), which can assemble into filamentous polymers (F-actin). These polymers undergo a continuous treadmilling process (Figure 1A; see e.g., Pollard et al., 2000; Mogilner and Edelstein-Keshet, 2002; Bennett et al., 2011 for details): G-actin with bound ATP is added preferentially to the barbed (+) ends of the filament (see for example added monomer marked with P in Figure 1A), while at the pointed (−) end older actin monomers of the filament are mostly depolymerized. Thus, actin filaments are polar structures with one end growing more rapidly than the other. This asymmetry between barbed and pointed end is further strengthened when the ATP bound at actin filaments hydrolyzes to ADP, which promotes disassembling of the pointed ends by severing proteins, such as cofilin (D in Figure 1A), when the pointed end is in an uncapped state (U in Figure 1A). Following this, the disassembled cofilin-ADP-actin dissociates to cofilin and an ADP-actin monomere and finally, profilin catalyzes the exchange of ADP to ATP and the resulting ATP-actin is again available for the polymerization process at the barbed end (omitted in Figure 1A). Additional to this treadmilling process, complexes, such as Arp2/3 can induce branching of a filament whose two daughter-filaments have uncapped barbed ends (B in Figure 1A) and capped minus ends. Moreover, barbed ends can become unable to polymerize G-actin due to capping proteins (C in Figure 1A).

Although the treadmilling process in dendritic spines occurs at different velocities, two distinctive pools of F-actin can be identified (Honkura et al., 2008): The static pool, which has a slow treadmilling velocity and is localized at the base of the spine head whilst the dynamic pool treadmills faster and is found at the tip of the spine head. Honkura et al. (2008) suggest that these pools have different functions: the static pool gives stability to the base of the spine, while the dynamic pool causes spine expansion due to the higher rate of actin polymerization resulting from the fast treadmilling velocity. Therefore, in this paper, we will focus on the dynamic pool. Interestingly, the fast treadmilling in this pool is not occurring uniformly distributed over the whole spine, but at discrete foci of actin polymerization (Frost et al., 2010). There are usually only a handful of those foci in one spine, which are well-separated from each other and can be identified by their increased polymerization rate. Because polymerization has been identified as the molecular mechanism responsible for spine shape fluctuations (Fischer et al., 1998), it can be assumed that these foci generate the main expansive force that underlies shape fluctuations, which are usually inhomogeneous and asymmetric. Although the role of actin in synaptogenesis and synaptic function has been thoroughly described (Cingolani and Goda, 2008; Hotulainen et al., 2009; Korobova and Svitkina, 2010; Basu and Lamprecht, 2018; Borovac et al., 2018), the exact role of actin spontaneous protrusions is still unknown, albeit found in the living mouse brain (Berning et al., 2012) and across different neuron types (Dunaevsky et al., 1999).

Mathematical models that link actin activity to such asymmetric spine fluctuations are, however, missing so far. Although models of the actin treadmilling process have been derived (Mogilner and Edelstein-Keshet, 2002) and adapted to the conditions in the dendritic spine (Bennett et al., 2011), they have not been connected to spine shape. To evaluate how shape is influenced by actin dynamics, one has to consider not only the forces created by the filaments, but also the counteracting forces from the lipid membrane that encloses the spine. Such models for force generation by actin filaments (Mogilner and Oster, 1996) and their interaction with the membrane have been derived and successfully applied to the movement of bacteria, cell motility (Mogilner and Oster, 2003; Rubinstein et al., 2009; Craig et al., 2015; for a review see Mogilner, 2006), and to explain dendritic spine maturation (Miermans et al., 2017). Yet, most of these models describe the dynamics of the cell shape based on density descriptions of the actin filaments or assume a homogeneous distribution of F-actin. However, considering the comparably small numbers of filaments within the spine (compare Korobova and Svitkina, 2010), a density description is not applicable. The homogeneity assumption, in turn, entails very regular and symmetric spine shapes, which are not observed in experiment (e.g., Fischer et al., 1998) and also are not consistent with the existence of actin polymerization foci.

Here, we present a model that considers heterogeneous actin dynamics caused by foci of actin polymerization. We use the forces generated by their treadmilling activity together with the counteracting forces from the membrane and the membrane-mediated coupling to other foci to derive a model of spine membrane shape fluctuations in 2D. Moreover, we extend the model to 3D to have a more realistic description of the spine and show that shape fluctuations behave similarly to those in 2D, suggesting that the 2D model can be used as a computationally more efficient surrogate. We show that the properties of spine fluctuations are strongly influenced by the dynamics of filament assembly constituting the determinants of the force generation by actin. The central finding of this study is that spine shape fluctuations can be fully explained by the effect that the small number of polymerization foci leads to a discretization of the outwards pushing-force direction, while their limited life time determines the temporal properties of these fluctuations. Thus, we can also show that spine area evolution can be predicted by the number of polymerization foci. Thus, this model provides the required biophysically detailed basis for future investigations of spine shape changes induced by synaptic plasticity.

## 2. Materials and Methods

### 2.1. Model

Based on the findings of Frost et al. (2010), we assume that the spine shape is determined by a small number of distinct foci of actin polymerization (gray filaments in Figure 1B), for which the processes of treadmilling, branching, and capping of the filaments are modeled individually (see section 2.1.2). As a consequence, each focus can have multiple barbed ends generating forces that push the membrane outward (see section 2.1.4, red arrows in Figures 1B,C). These forces concur with the inward directed forces generated by the membrane's resistance against deformation (see section 2.1.5, green arrows in Figures 1B,C). If these forces are locally unbalanced, the membrane moves giving rise to shape fluctuations (transition from black to gray membrane shape in Figure 1C). To simulate this interaction of membrane shape and forces, we use discrete time-steps and the finite elements method. In particular, the membrane is represented by a mesh of points (or vertices) for which geometrical properties, forces, and movements are calculated (see Supplementary Material).

#### 2.1.1. Membrane Mesh Initialization and Morphological Constraints

As stated above, we represent the membrane enclosing the spine by a mesh of vertices *k* ∈ {1, 2, ...*n*_*vertices*_} described by their (two-dimensional) position vectors **x**^*k*^ = (*x*^*k*^, *y*^*k*^), 1 ≤ *k* ≤ *n*. Upon initialization, a polygonal approximation for a circle with radius *r*_*s*_ and centered at the origin of the x-y plane is created. As in this study we focus on the shape fluctuations of mature spines, we implement two major morphological constraints:

On the one hand, the spine neck of mature spines typically contains heavily interlinked actin bundles which are rather stable and have a much slower treadmilling velocity than those in the spine tips (Okamoto et al., 2004). Along this line, the spine neck width is largely stable on the here considered timescale of hours (Tønnesen et al., 2014). Therefore, we fix the location of mesh-points at the neck throughout the whole simulation. We establish those fixed mesh points during the mesh initialization by selecting all points **x** = (*x, y*) with *y* ≤ *h*_*neck*_ and fixing them to (*x, h*_*neck*_). Here we define *h*_*neck*_ as the value of *y* where *x* = *r*_*neck*_ and *y* < 0.

On the other hand, the movement of the post-synaptic density (PSD) is constrained as it is heavily interlinked with the presynaptic site. Also, the PSD size on unstimulated spines is conserved over the timescale of hours (Meyer et al., 2014). Therefore, we fix the mesh-points (*x, y*) with *y* ≥ *h*_*PSD*_ to (*x, h*_*PSD*_), where *h*_*PSD*_ is the value of *y* where *x* = *r*_*PSD*_ and *y* > 0. Hence, the resulting initial shape resembles a flat disk. A schematic depiction of this process is given in Figure S1. Note that because the number of synaptic receptors is correlated with PSD size, the assumption of fixed PSD size implies no change in synaptic current.

#### 2.1.2. Actin Dynamics at Individual Foci

We adapt the stochastic model proposed by Bennett et al. (2011) for F-actin dynamics of the actin dynamic pool in the spine head. Because we are concerned about the spontaneous spine shape fluctuations, we only simulate the treadmilling process of Bennett et al. (2011) in which G-actin is polymerized at the uncapped barbed ends in every time-step of length Δ_*t*_. In addition, the following processes can occur at each actin filament:

- Uncapped barbed ends branch by including an Arp2/3 molecule and give rise to a new filament with a probability Δ_*t*_γ_*branch*_(*t*).
- Uncapped barbed ends are capped by a capping protein with a probability Δ_*t*_γ_*cap*_. Because polymerization is not possible when a barbed end is capped, such barbed end does not generate force. As uncapping occurs very seldom, these filaments are eliminated from the simulation.
- Capped minus ends are uncapped with a probability Δ_*t*_γ_*uncap*_.
- Uncapped minus ends are severed with a probability Δ_*t*_γ_*sever*_, which leads to the removal of the respective filament.

Note that these events are necessary so that the length and concentration of F-actin are within a biologically plausible range. Otherwise, filaments will grow and live indefinitely. In Bennett et al. (2011), the branching rate for a filament γ_*branch*_(*t*) depends on the total number of barbed ends *B*(*t*) in the spine at time *t*, $\gamma_{branch}\left(t\right)={\hat{\gamma }}_{branch}\left(t\right)/B\left(t\right)$. However, in our model we assume that there are discrete foci of actin polymerization. Hence, we adapt the branching rate to be proportional to the local number of barbed ends *B*^*i*^ at each focus *i*, thus $\gamma_{branch}^{i}\left(t\right)={\hat{\gamma }}_{branch}\left(t\right)/B^{i}\left(t\right)$.

Moreover, actin dynamics in our model are embedded in a 2D membrane that approximates the dendritic spine morphology instead of one straight line used by Bennett et al. (2011) in which the barbed ends at the membrane branch freely at a rate ${\hat{\gamma }}_{branch}\left(t\right)$ proportional to a constant treadmilling velocity *v*_*T*_(*t*). However, the cell membrane imposes a resistance to filament assembly and thus, decreases this rate: if the membrane resistance is high, then the filament will be less likely to branch. In our model, this membrane resistance depends on the local spine geometry. Hence, following Mogilner and Edelstein-Keshet (2002) we assume that the branching rate depends on the treadmilling velocity *v*_*T*_(*t*) = ϕ*k*_*on*_δ*a* where δ is the length of an actin monomer, *k*_*on*_ the barbed end monomer assembly rate, and *a* the concentration profilin-ATP-actin available for polymerization. As we are not modeling plasticity related changes in this study, we can consider *a* as a constant (see Supplementary Material). This free polymerization velocity is attenuated due to a counteracting membrane force according to the Brownian ratchet theory (Mogilner and Oster, 1996; Footer et al., 2007), which takes into account the absolute temperature *T*, the Boltzmann's constant *k*_*B*_ and the force ${\text{F}}_{mem}^{i}\left(t\right)$ working against polymerization which is generated by the membrane at the *i*th focus center. Thus, the branching probability at each focus is given by

(1) $$\begin{array}{l} \gamma_{branch}^{i}\left(t\right)=\phi k_{on}\delta a\exp\left(-\frac{||{\text{F}}_{mem}^{i}\left(t\right)||\delta }{k_{B}TB^{i}\left(t\right)}\right)\frac{1}{B^{i}\left(t\right)}. \end{array}$$

Note that this membrane-force-dependency of the branching rate generates a feedback between the number of barbed ends and membrane shape.

In this study, we are not interested in the geometrical configuration of the actin filaments but rather in the amount of force generated by actin polymerization. Therefore, we characterize F-actin by the states (capped/uncapped) of its barbed end (normally uncapped) and pointed end (normally capped or bound to a Arp2/3 complex) instead of explicitly simulate each filament and how it grows in space as in Bennett et al. (2011). Such filament states change when a branching, capping or severing event occur in the model described above, i.e., when a random number drawn for this filament falls below the indicated probability. We iterate through all filaments with uncapped barbed ends within the active actin polymerization foci and the above processes in the indicated order. Afterwards, the remaining uncapped barbed ends in each polymerization focus *i* are counted and their number *B*^*i*^ is used to calculate the expansive force exerted by that focus (see section 2.1.4). Figure 1D shows an exemplary temporal evolution of the active filaments in one of the polymerization foci, where all of these processes occur. The rate values are stated in Table 1.

**Table 1:** Model parameter values.

**Symbol**	**Unit**	**Definition**	**Value**	**References**
Δ_*t*_	s	Length of the time-step	1/8	Frost et al., 2010 (fitted as in Mogilner and Edelstein-Keshet, 2002)
δ_*s*_	μm	Target length of and edge	0.03	Fitted for numerical accuracy (see text)
*r*_*s*_	μm	Initial spine radius	0.5	Tønnesen et al., 2014; Miermans et al., 2017
*r*_*neck*_	μm	Neck radius	0.0995	Tønnesen et al., 2014; Miermans et al., 2017
*r*_*PSD*_	μm	PSD radius	0.3571	Meyer et al., 2014
*n*_*f*_0__	1	Initial number of nucleation points	4	Frost et al., 2010
γ_*cap*_	s^−1^	Barbed-end capping rate	1	Bennett et al., 2011
γ_*uncap*_	s^−1^	Uncapping rate for—ends	1/30	Bennett et al., 2011
γ_*sever*_	s^−1^	Depolymerization/Severing rate of—ends	1	Bennett et al., 2011
γ_*f*_	s^−1^	Nucleation rate of new actin focus of activity	0.1	Basu and Lamprecht, 2018, see text
*a*	μM	Concentration of profilin-ATP-actin at steady state	3.8	Bennett et al., 2011, see Supplementary Material
ϕ	μm^−2^	Proportionality constant	75	–
*k*_*on*_	μM^−1^s^−1^	Barbed-end monomer assembly rate constant	11.6	Mogilner and Edelstein-Keshet, 2002
δ	μm	Length of an actin monomer	0.0022	Mogilner and Edelstein-Keshet, 2002
*k*_*B*_*T*	pNμm	Thermal energy	0.0041	Mogilner and Edelstein-Keshet, 2002
*P*	pNμm^−2^	Difference between internal and external pressure	85.7143	Young-Laplace law see Deserno, 2015
τ	pNμm^−1^	Surface tension	15	Pontes et al., 2013
κ	pNμm	Bending modulus	0.18	Pontes et al., 2013
α	pN	Strength of filament influence	3.8	Miermans et al., 2017
σ	1	Extend of filament influence	0.3	–
ζ	μm^2^s^−1^pN^−1^	Strength of force update	0.002	–
λ	μm	Nucleation distance parameter	0.025	–

#### 2.1.3. Foci Generation and Removal

Note that the activity of a focus is determined by its uncapped barbed ends, which can only emerge from other uncapped barbed ends due to branching; hence, foci naturally become inactive and removed as soon as they have no uncapped barbed ends left. Therefore, mechanisms for generation of new foci are necessary. Frost et al. (2010) observed that the discrete actin polymerization foci locate mostly at the spine tip but also through out the spine. The majority of their F-actin is shorter than 200 nm and reaches a peak density within 300 nm of the PSD center. Moreover, Frost et al. (2010) noted that the dynamics of F-actin at these foci must be regulated near the membrane due that the branching protein Arp2/3 concentrates within 100 nm of the spine membrane whilst the filament severing protein cofilin concentrates within 200 nm of the membrane. Furthermore, data from Rácz and Weinberg (2008) show that Arp2/3 complex is mainly distributed in a doughnut-shaped domain within the spine that could represent a zone where F-actin branches and generates the forces necessary for membrane protrusion. Taken all together, we assume that actin polymerization foci must initiate near to the membrane and PSD so that filaments conserve their small size and co-locate with the branching proteins.

Therefore, in our model the nucleation of a new actin polymerization focus *i* is implemented in two steps: First, a two dimensional nucleation position denoted by a vector ${\text{X}}_{n}^{i}$ (see Figure 1C) is selected in the following way: To simultaneously account for the above geometrical restrictions and the asymmetrical form of the spine head, we generate a set of 1,000 uniformly distributed candidate points inside the spine. From this candidate set, we remove all points that are not within a distance of 0.1μm from the membrane and that are within 0.1μm from the PSD. Then, one of the remaining *n*_*cand*_ points *j* is selected with probability $p_{j}={\text{e}}^{-d_{j}/\text{λ}}{\left(\sum_{l=1}^{n_{cand}}{\text{e}}^{-d_{l}/\text{λ}}\right)}^{-1}$, which depends on its distance from the PSD *d*_*j*_ via a scale parameter λ. For λ → 0^+^ nucleation near to the PSD is favored whereas for λ → ∞ the distance to the PSD has no influence.

Second, the primary nucleation direction is randomly selected as the vector pointing from ${\text{X}}_{n}^{i}$ to one of the membrane points that are within 0.1μm. The position of the selected membrane vertex *k* is referred to as the center of the focus ${\text{X}}_{c}^{i}:={\text{x}}^{k}$. As the foci are relatively short-lived, we assume that this direction does not change over the lifetime of the focus.

#### 2.1.4. Actin-Generated Force

As in Mogilner and Oster (2003), we take the propulsive force generated by actin polymerization to be proportional to the number of uncapped barbed ends within each focus. Here, such force is assumed to be acting at the center of an actin polymerization focus ${\text{X}}_{c}^{i}$ and to extend laterally to the nearby vertices. When a branching event occurs, a new filament extends at an angle of 70° from the branched filament. Here, instead of explicitly modeling each filament, as in Bennett et al. (2011), we assume that a continuously changing amount of short-lived filaments with uncapped barbed ends at each focus generates a force that distributes symmetrically. Hence, the force contributions can be summed into a Gaussian Kernel around the focus center, given by

(2) $$\begin{array}{l} W(x)=\frac{\alpha }{\sigma \sqrt{2\pi }}{\text{e}}^{-\frac{x^{2}}{2\sigma^{2}}}, \end{array}$$

with an amplitude α and standard deviation σ. Then, the resulting force vector at the vertex *k* (located at **x**^*k*^) is proportional to the number of barbed ends in the focus and is given by

(3) $$\begin{array}{l} {\text{F}}_{fil}\left({\text{x}}^{k}\right)=\underset{i\in 1,2,..n_{f}}{\sum }W\left(||{\text{x}}^{k}-{\text{X}}_{c}^{i}||\right)B^{i}\left(t\right){\text{V}}^{i,k}, \end{array}$$

with *n*_*f*_ being the number of currently active actin foci with *B* barbed ends and ${\text{V}}^{i,k}=\frac{{\text{x}}^{k}-{\text{X}}_{n}^{i}}{||{\text{x}}^{k}-{\text{X}}_{n}^{i}||}$ the normalized direction vector of the force from focus *i*. In this way, the force exerted by actin is spatially extended proportionally to the number of barbed ends at each focus.

#### 2.1.5. Membrane Force

Biological lipid membranes, such as the one confining the spine head, are composed of single molecules that self-assemble into stable fluid films of macroscopic lateral scales with lateral dimensions greatly exceeding their thickness (Deserno, 2015). Therefore, modeling approaches consider the membranes as two-dimensional elastic continuum (Guckenberger and Gekle, 2017) (described by a manifold Γ) in which any deviation of the equilibrium shape increases the membrane energy and induces response forces that return the membrane to its equilibrium (Krüger, 2012). Generally, the bending contributions to this energy are described by the Helfrich free energy (Helfrich, 1973), for which constrains of size and total surface conservation are often added. Hence, the membrane energy is given by

(4) $$\begin{array}{l} E_{mem}=P\Omega +\tau S+2\kappa \int_{\Gamma }H^{2}\text{d}s \end{array}$$

where the membrane's physical properties are characterized by the difference between internal and external pressure *P*, the line tension (or surface tension in 3D) τ, and the bending modulus κ. Ω is the area enclosed by the membrane (or volume in 3D), *S* is the boundary length (or surface area) and *H* is the mean curvature. The membrane force vector ${\text{F}}_{mem}\left({\text{x}}^{k}\right)$ at vertex *k* is given by

(5) $$\begin{array}{l} {\text{F}}_{mem}\left({\text{x}}^{k}\right)=-\frac{\partial E_{mem}}{\partial {\text{x}}^{k}}. \end{array}$$

On our discrete mesh, the geometrical properties Ω, *S*, and *H*, and hence, the resulting force can be approximated for each vertex by taking its next neighbors in the mesh into account (see Supplementary Material). Note, however, that the approximations of the geometrical properties are only valid when the mesh is dense enough. Therefore, when the vertices move too far apart from each other, we refine our mesh (remeshing, see section 2.2).

Together with the fixed spine neck and PSD vertices, the membrane force gives rise to a characteristic “resting shape,” to which the spine converges in the absence of other forces to minimize area, length and curvature (see e.g., **Figure 4A** for resting shapes resulting for different PSD-sizes).

#### 2.1.6. Membrane Movement

In the presence of both actin and membrane generated forces, the spine shape is determined by a balance between them. If the forces are unbalanced at one of the mesh vertices, they will generate a movement of that vertex and deformation of the membrane. For simplicity, we assume that the motion of the vertex *k* is proportional to the net force with a proportionality constant ζ. Thus, the displacement of vertex *k* in time is given by

(6) $$\begin{array}{l} \frac{\text{d}{\text{x}}^{k}}{\text{d}t}=\varsigma \left({\text{F}}_{mem}\left({\text{x}}^{k}\right)+{\text{F}}_{fil}\left({\text{x}}^{k}\right)\right). \end{array}$$

This equation is implemented in discrete time-steps using a classical Runge-Kutta algorithm, in which we keep ${\text{F}}_{fil}^{k}$ constant during the whole calculation step. However, the interaction between neighboring membrane points can still give rise to diverging oscillations. Therefore, if the membrane displacement in a single time-step exceeds a certain displacement tolerance (*d*_*tol*_ = 0.0001μm), we split this time-step in two intervals and calculate the displacement of all membrane vertices in each of them until the displacement is smaller than the tolerance.

### 2.2. Simulation

#### 2.2.1. Individual Foci

First, a single actin polymerization focus is simulated using the Monte Carlo model (section 2.1.2) with fixed ||**F**_*mem*_|| in Equation (1). The focus is initialized with different numbers of barbed ends (between 1 and 20) and simulated until all barbed ends have vanished. Hereby, the number of barbed ends in each time-step as well as the lifetime of the focus are tracked. In order to compare the outcomes of these simulations with theoretical expectations, we investigate the dynamics of the barbed ends in a deterministic framework where we take *B* as a continuous quantity. For this, we derive the rate equations assuming that a focus has *B* filaments with barbed ends that classify into two types according to the state of its minus end. Here, *m*_*c*_ denotes the filaments with capped and *m*_*u*_ filaments with uncapped minus end, and the deterministic dynamics of *B* in a focus is given by

(7) $$\begin{array}{l} \frac{\text{d}m_{u}}{\text{d}t}=\gamma_{uncap}m_{c}-\gamma_{sever}m_{u}-\gamma_{cap}m_{u},\\ \frac{\text{d}m_{c}}{\text{d}t}={\hat{\gamma }}_{branch}\left(t\right)-\gamma_{uncap}m_{c}-\gamma_{cap}m_{c},\\ \;B=m_{u}+m_{c}. \end{array}$$

As ${\hat{\gamma }}_{branch}\left(t\right)$ in turn depends on *B*, these equations are highly non-linear and have been solved for their stationary state numerically. Note that the steady state of such deterministic system should match the mean value of our stochastic simulations.

#### 2.2.2. 2D Model

Simulations are performed in MATLAB on a desktop computer. Table 1 contains the parameters used in the simulations, unless stated otherwise. We first initialize the mesh by tracing a circle with equidistant vertices of δ_*s*_, then the vertices of PSD (neck) are fixed as described in section 2.1.1. Subsequently, we simulate an initialization period in which the mesh points **x**^*k*^ move considering only the force generated by the membrane (see Figure S1). In such simulation, the force generated by the membrane is computed for each vertex (following to the calculations given in the Supplementary Material). Then the vertices move according to Equation (6), except those belonging to the PSD or neck that we fixed when generating the initial shape. During this initialization period the spine shape shrinks until it reaches a stable configuration, which we refer to as the resting shape.

As discussed above, the finite elements approximations of the geometrical properties are only valid when the mesh is dense enough. If the vertices move too far apart from each other these properties are lost, and therefore, the mesh has to be redefined. Thus, we perform remeshing at each time-step by calculating recursively the distance between two neighboring vertices and remove one if the distance between them is below *d*_*min*_ = (3/5)δ_*s*_ or add a new vertex in between if the distance is above *d*_*max*_ = (4/3)δ_*s*_. Hence, for a mesh with vertices $\left\{{\text{x}}^{1},\ldots ,{\text{x}}^{k},{\text{x}}^{k+1},\ldots ,{\text{x}}^{n_{vertices}}\right\}$, if $\left\|x^{k},x^{k+1}\right\|>d_{max}$ then **x**^*new*^ = (**x**^*k*^ + **x**^*k*+1^)/2 and the new mesh is given by $\left\{{\text{x}}^{1},\ldots ,{\text{x}}^{k},{\text{x}}^{new},{\text{x}}^{k+1},\ldots ,{\text{x}}^{n_{vertices}}\right\}$. Note that the order of the mesh persists despite the addition or deletion of vertices that changes the size of the mesh *n*_*vertices*_.

After finding the resting shape configuration of the dendritic spine, we include actin dynamics and forces in the simulation (sections 2.1.2 and 2.1.4). For this, initially, *n*_*f*_0__ actin polymerization foci are inserted as described in section 2.1.3 and the generation of new foci is enabled. Note, that the indicated simulation times start after the initialization phase. During the simulations (**Figure 3**) we track the spine shape by saving the mesh regularly as well as the spine area, which is recorded every time-step. To assess the influence of different model parameters (**Figures 4**–**10**), we perform one 90 min simulation for each parameter value and determine the mean, standard deviation and auto-correlation function of the spine area fluctuations. Moreover, we evaluate the distribution over the assumed values of the number of foci and barbed ends and the mean lifetime of polymerization foci. We then perform fifteen 15-min-simulations with different initial polymerization foci to obtain statistics for different initial conditions, estimate their uncertainty, and test whether values vary significantly.

#### 2.2.3. 3D Model

Simulations in 3D are performed as in 2D, albeit with some changes in the analytical calculations for the membrane force. For example, the curvature calculation of a vertex in 2D only considers that and the next adjacent vertices, whilst in 3D this calculation is a function of the six neighbors of that vertex (see Supplementary Material for more details). Therefore, simulations in 3D are more complex.

In the 3D case, the mesh is initialized using the MATLAB function icosphere.m (Ward, 2015) which generates a unit geodesic sphere that we multiply by *r*_*s*_. Additionally, in 3D the mesh has to be isotropic and conserve the number of neighbors of each vertex at each time-step to maintain the geometrical properties of the finite elements approximation. Thus, remeshing is implemented using the MATLAB function remeshing.m (Helf, 2015), that is based on OpenMesh (Botsch et al., 2002; Computer Graphics Group, 2018). The target edge length is set to δ_*s*_ and three iterations are performed each time. To compare the 3D shapes with the 2D simulations we do a two-dimensional projection of the three-dimensional mesh. For this, we project all points to the x-z-plane and trace a boundary around the projected points using the MATLAB function boundary.m. Likewise, we project to the y-z-plane to compare the two 2D projections of the same spine.

## 3. Results

### 3.1. Individual Polymerization Foci Have Finite Lifetime Depending on the Membrane Force

**Figure 2:**
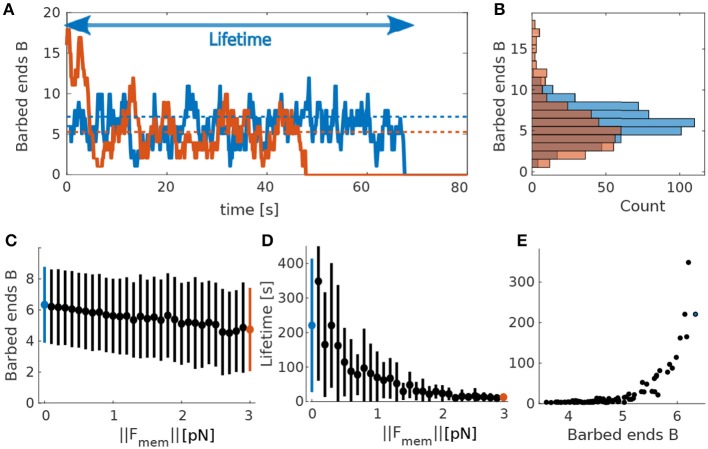
Actin polymerization focus. **(A)** Evolution of barbed ends over time at a single actin polymerization focus. Blue solid line corresponds to ||**F**_*mem*_|| = 0pN and orange solid line to ||**F**_*mem*_|| = 3pN. Dotted lines indicate theoretical mean values calculated using the deterministic model for barbed ends in Equation 7. Colors correspond to the different values of ||**F**_*mem*_||. Arrow indicates the lifetime of the focus corresponding to the blue curve. **(B)** Occurrence frequency (x-axis) of each number of barbed ends (y-axis) over all time-steps (of length Δ_*t*_) in **(A)**. Colors as in **(A)**. **(C)** Mean and standard deviations of mean number of barbed ends *B* over 50 simulations each for different values of ||**F**_*mem*_||. **(D)** Same for focus lifetimes. Blue and red dots mark the force values used in **(A,B)**. **(E)** Mean lifetime over mean number of barbed ends for 50 simulations varying force values.

In order to understand the highly non-linear interaction between actin dynamics and membrane force, we first simulated the model for a single actin polymerization focus with a constant counteracting membrane force **F**_*mem*_ in Equation (1). We then tracked the time course of the number of barbed ends in our Monte-Carlo model (Figure 2A). During these simulations the number of barbed ends changes on the scale of 100 ms, meaning that there is a fast turnover of barbed end, which justifies our assumption of force kernel (section 2.1.4). Moreover, the number of barbed ends fluctuates around a mean value (dashed lines in Figure 2A) on a timescale of seconds. This mean value depends on the counteracting force: If **F**_*mem*_ increases, the mean number of barbed ends decreases (blue and orange dashed lines in Figures 2A–C). Importantly, the foci have non-zero probability to transit to *B* = 0. At that point no barbed end can be generated by branching anymore and the focus is dynamically dead. As a result all foci have limited lifetime. We also tracked this lifetime at different counteracting forces and find that it decreases when increasing the force (see Figure 2D). We finally evaluated the relation between the mean number of foci and the lifetime for varying forces and find that foci with more barbed ends live longer (Figure 2E), as expected from the above reasoning.

The dependency of the mean number of barbed ends (Figure 2C) and the lifetime of a focus (Figure 2D) on the membrane force can be explained by the fact that increasing **F**_*mem*_ decreases the branching rate (see Equation 1). Accordingly, the mean number of barbed ends is smaller and the distribution is shifted toward smaller numbers of *B* (Figures 2B,C). This increases the probability of being at *B* = 1, and, in turn, the probability to reach zero barbed ends. Consequently, the lifetime of these actin polymerization foci decreases when increasing the counteracting force. This relation between membrane force and focus lifetime indicates that the spine shape, which determines the membrane force, influences magnitude and the temporal properties of the shape fluctuations.

### 3.2. Shape-Fluctuations of the Spine

**Figure 3:**
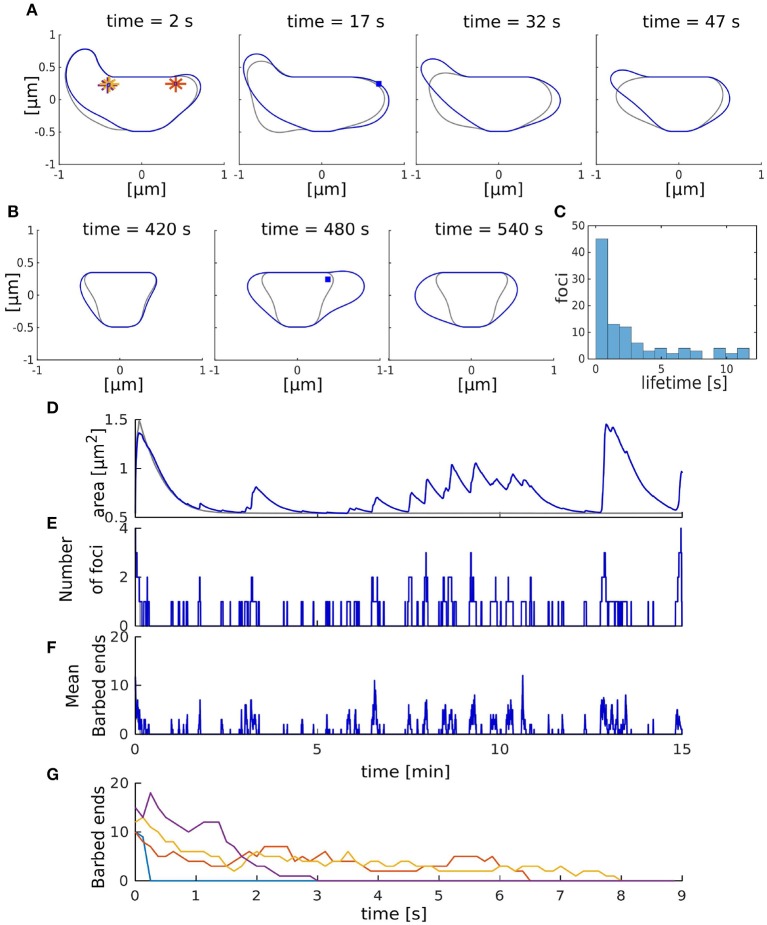
Shape fluctuations of the spine. **(A)** Snapshots of the spine head shape with (blue line) and without (gray line) nucleation of new actin polymerization focus taken every 15 s. Asterisk correspond to the initial nucleation positions **X**_*n*_ and blue squares indicate nucleation positions of the active foci at the indicated time. **(B)** Same as **(A)** but taken every 60 s. **(C)** Occurrence frequency of foci lifetime over all time-steps (of length Δ_*t*_) in the simulation with nucleation. **(D–F)** Evolution of the spine area **(D)**, number of foci **(E)**, and mean number of barbed ends **(F)** over time. Gray and blue lines represent the simulation without and with nucleation of new foci, respectively. **(G)** Evolution of the number of barbed ends in each actin focus (color-coded) in the simulation without nucleation of new foci.

In the next step, we studied the interplay between the membrane and actin forces. For this, we simulated the full model with multiple actin polymerization foci distributed within a spine head (Figures 3A,B). We initialized the model with four of such foci that push the spine membrane outward. However, as the lifetimes of foci (Figures 3C,G) are much shorter than the lifetime of spines, which can persist over months (Holtmaat et al., 2005), we introduced a focus creation mechanism (i.e., nucleation of new actin polymerization foci, see section 2.1.3) where new foci are created at the beginning of each time-step at a rate γ_*f*_. As it is not clear from experimental data where such nucleations happen, we also introduced a distance parameter λ which allows us to scale continuously between focus nucleation everywhere within the spine and nucleation close to the PSD. The influence of these parameters on the emerging shape fluctuations will be investigated in detail later (see section 3.3.5). Without this nucleation mechanism, all foci quickly reach zero barbed ends (in <9 s in Figure 3G) and the spine returns to the resting shape (gray line in Figure 3B). Moreover, spine area fluctuations also cease (gray line in Figure 3D).

Figures 3A,B (blue line) show the resulting shape dynamics of the spine in our model. The proposed nucleation mechanism together with the short lifetimes of individual foci allows the spine to have different asymmetric shapes over time, which are qualitative similar to the experiments (Fischer et al., 1998). Note that during the depicted time interval, several foci have died out and several others have been nucleated (Figure 3E). Moreover, the mean number of barbed ends at these foci is continuously fluctuating (Figure 3F). In general, we observed that spine area increases when several foci are active at the same time or when a focus is long-lived (Figures 3D,E). Thus, shape and area fluctuations of a spine are the result of the transiently active foci working against the membrane. In particular, they are generated by the stochasticity of the molecular dynamics of actin filament assembly, which eventually leads to the die-out of foci. Therefore, it can be expected that the molecular dynamics as well as the mechanics through which they interact with the membrane will have a major impact on the emerging fluctuations.

### 3.3. Influence of Model Parameters

In order to better understand how spine size fluctuations are affected by the dynamics of actin and the interplay between forces generated by actin polymerization foci and spine membrane, we investigated the effect of varying multiple model parameters. For this, we used the parameters in Table 1 and increased or decreased the value of one selected parameter at a time.

#### 3.3.1. Size of the Post-synaptic Density

**Figure 4:**
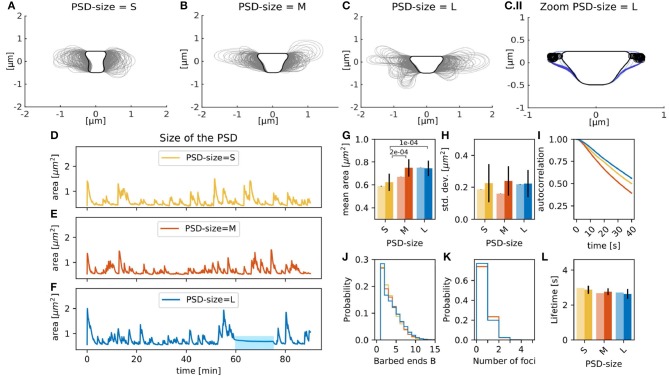
Spines with varying PSD size. Spine shapes (gray lines, plotted every 15 s) over 90 min of simulation for **(A)** small, **(B)** medium, and **(C)** large sized PSDs. Black line corresponds to the resting shape. **(C.II)** Shapes of the large PSD-size spine between min 60 and 70 (color-coded from dark to light blue). Black open circles correspond to the nucleation points. **(D–F)** Evolution of the spine area over time. **(G)** Temporal mean over area in 90 min simulation (pale bars, errors are standard deviations over 50 bootstraps) and average of temporal means over fifteen 15 min simulations (saturated color, errors are standard deviations of the mean) over different PSD sizes. The *p*-value for significant Welch-tests is indicated. **(H)** Same as **(G)** but for standard deviation of the area fluctuations. **(I)** Autocorrelation functions for the area fluctuations in the 90 min simulations. **(J)** Histogram of the mean number of barbed ends at the polymerization foci at each time-step of the 90 min simulation. **(K)** Histogram of the active actin polymerization foci. **(L)** Mean lifetime of a focus, color-coded as in **(G)**.

Experimental studies show that there is a strong correlation between spine volume and PSD size (Arellano et al., 2007; Meyer et al., 2014). Therefore, we tested if this correlation holds in the presence of spontaneous fluctuations by changing the size of the PSD (*r*_*PSD*_, see section 2.1.1), which affected the size, and thus, also the area of the resting spine shape. For example, when the radius of the PSD was enlarged to *r*_*PSD*_ = 0.4330, the distance between PSD and neck was also affected, which altered the resting shape of the spine (Figures 4A,C, black line). Accordingly, the mean spine area, evaluated over 90 min simulations of individual spines, increased with the PSD size (Figure 4G, pale bars). To test whether this tendency is significant, we performed a Welch-test comparing the mean spine areas in fifteen 15 min simulations for each PSD size (Figure 4G, full colored bars). We found that the small PSD size (set to *r*_*PSD*_ = 0.2179) gives rise to significantly smaller mean areas.

Figures 4D,F shows that area fluctuations behave differently when the PSD size varies. To quantitatively describe such fluctuations, we used the standard deviation and autocorrelation functions. A small standard deviation indicates that spine area fluctuations tend to be close to the mean, whilst high values indicate larger area fluctuations. The autocorrelation function shows the area correlation with itself at different time points. Therefore, if the autocorrelation function decays fast, then the area fluctuation is correlated to itself only for a short time, indicating that the area fluctuations occur more rapidly. Thus, the area standard deviation accounts for the size of area fluctuations and the slope of the autocorrelation function for their speed. Using these functions, we find that the medium PSD-size spine from the 90 min simulation shows a smaller area standard deviation than the 90 min simulations of spines with different PSD-size (Figure 4H, pale bars), multiple simulations show that the area standard deviations are similar. We found that the area of spines with large PSD-size is temporally longer correlated to itself, indicating that the fluctuations occur more slowly (Figure 4I). One reason for this might be that the membrane forces typically decrease for larger spines such that the decay back to the rest shape happens more slowly. Interestingly, the autocorrelation for spines with medium PSD size decays even faster than that for small PSD size. This may be due to the fact that the actin polymerization foci in spines with smaller PSD tend to last longer (Figure 4L). Taken all together, the correlation between the spine and PSD size find in experiments hold in our model with spontaneous shape fluctuations. Interestingly, the size of these fluctuations was similar despite PSD size due that the number and lifetime of the actin polymerization foci were not significantly affected by the PSD size.

The 90 min simulation of the spine with a large PSD-size also exhibits a period (between min 60 and 70) where the spine area fluctuations cease (blue bar in Figure 4F, spine shapes in Figure 4C.II). Although new foci are nucleated during this period, changes in spine shape are minor because new foci randomly nucleate nearby each other (locations marked by circles in Figure 4C.II). Moreover, the curvature near these foci is large, such that the force generated by the membrane is higher decreasing the branching rate; and hence, the lifetime of those foci.

#### 3.3.2. Branching Rate Amplitude ϕ

**Figure 5:**
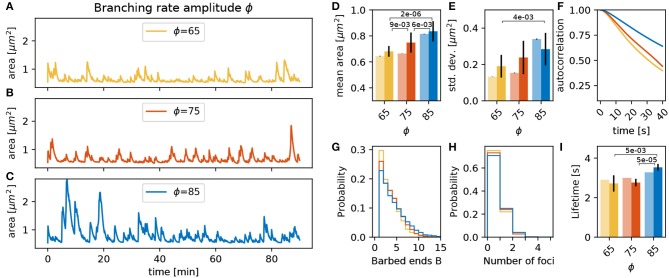
Varying the branching rate amplitude ϕ. **(A–C)** Evolution of the spine head area over time for different values of ϕ: **(A)** ϕ = 65, **(B)** ϕ = 75, and **(C)** ϕ = 85. **(D)** Temporal mean of the spine head area. Light bars correspond to values from single 90 min simulations (standard deviations obtained from 50-fold bootstrap). Full colored bars and errors correspond to the mean and standard deviation over fifteen 15 min simulations. The *p*-value for significant Welch-tests is indicated. **(E)** Standard deviation of the spine head area over time. **(F)** Autocorrelation functions for the area fluctuations in the 90 min simulations. **(G)** Relative frequency of the mean number of barbed ends per focus over all simulation time-steps from **(A–C)**. **(H)** Relative frequency of the number of actin polymerization foci. **(I)** Mean lifetime of a focus.

The branching rate of actin polymerization foci γ_*branch*_ in Equation (1) is scaled by an amplitude ϕ. Three simulations with different values of ϕ over 90 min, as well as the fifteen 15 min repetitions with different initial conditions, show that an increase of ϕ enlarges the mean spine head area significantly (compare orange and blue bars in Figure 5D, *p*-value for significant Welch-tests indicated). The 90 min simulations (Figures 5A,B) show that a decrease of ϕ induces a small decrease in spine mean area (light bars in Figure 5D). However, the fifteen 15 min simulation show a larger decrease (full bars in Figure 5D). This relation between the mean area and ϕ can be explained by the fact that spines with a larger value of ϕ also have more barbed ends at the actin polymerization foci (Figure 5G). Due to this increased number of barbed ends, the polymerization foci of those spines tend to last longer (Figure 5I). As a consequence, there are more actin polymerization foci in spines with a larger branching rate amplitude (Figure 5H), which push the membrane outwards and increase the area. A similar picture emerges for the magnitude of the fluctuations measured by the standard deviation (Figure 5E) of the area as well as for the timescale of the autocorrelation decay (Figure 5F). Especially for large values of ϕ we observe a significantly larger standard deviation (Figure 5E) and a slower autocorrelation decay (Figure 5F). This fits well with the idea that the polymerization foci are more long lived; and therefore, push the membrane outward for longer times leading to larger area deviations. We conclude that an increase of ϕ enlarges the mean, the standard deviation and the autocorrelation decay timescale of the spine area fluctuations due to an increase of the lifetime of actin polymerization foci. However, a decrease in ϕ by the same magnitude does not affect the spine area to the same degree, which highlights that the underlying processes are subject to non-linear interactions.

#### 3.3.3. Lateral Extent of Actin Filament σ

**Figure 6:**
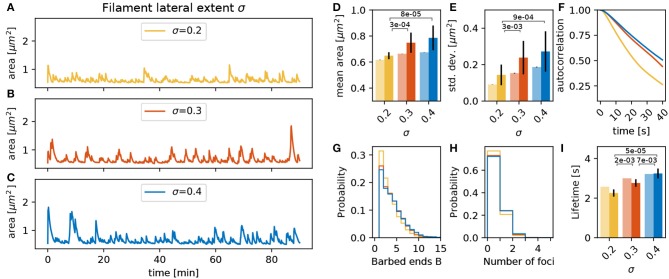
Varying the filament lateral extent σ. **(A–C)** Evolution of the spine head area over time for different values of σ: **(A)** σ = 0.2, **(B)** σ = 0.3, and **(C)** σ = 0.4. **(D)** Temporal mean of the spine head area. Light bars correspond to values from single 90 min simulations (standard deviations obtained from 50-fold bootstrap). Full colored bars and errors correspond to the mean and standard deviation over fifteen 15 min simulations. The *p*-value for significant Welch-tests is indicated. **(E)** Standard deviation of the spine head area over time. **(F)** Autocorrelation functions for the area fluctuations in the 90 min simulations. **(G)** Relative frequency of the mean number of barbed ends per focus over all simulation time-steps in **(A–C)**. **(H)** Relative frequency of the number of actin polymerization foci. **(I)** Mean lifetime of a focus.

Besides the number of barbed ends at the actin polymerization foci, the actin generated forces and the resulting deformations of the membrane are also determined by the lateral spatial extent of actin filaments (σ in Equation 2). When the lateral extent is small, the width of the bump pushing the membrane forward generated by the focus is narrow. The shape of this bump has a direct effect on the geometrical properties of the spine membrane around the focus. For example, a narrow protrusion has a greater curvature, which produces an increase in ||**F**_*mem*_|| working against this deformation. This entails a decrease in the branching rate as well as in the mean number of barbed ends (Figure 6G) and leads to less active foci with a shorter lifetime (Figures 6H,I). This shorter lifetime implies that foci push the membrane for shorter time such that the variations in the spine area become smaller (Figure 6E) and decay faster (Figure 6F).

#### 3.3.4. Movement Speed ζ

**Figure 7:**
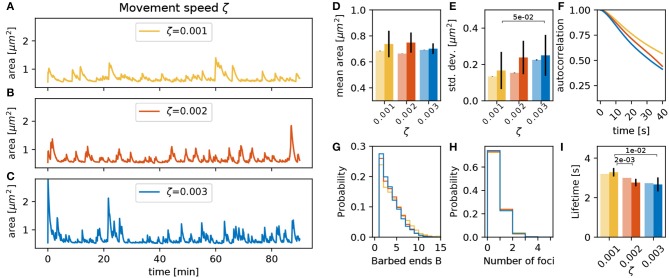
Varying the movement speed ζ. **(A–C)** Evolution of the spine head area over time for different values of ζ: **(A)** ζ = 0.001, **(B)** ζ = 0.002, and **(C)** ζ = 0.003. **(D)** Temporal mean of the spine head area. Light bars correspond to values from single 90 min simulations (standard deviations obtained from 50-fold bootstrap). Full colored bars and errors correspond to the mean and standard deviation over fifteen 15 min simulations. **(E)** Standard deviation of the spine head area over time. The *p*-value for significant Welch-tests is indicated. **(F)** Autocorrelation functions for the area fluctuations in the 90 min simulations. **(G)** Relative frequency of the mean number of barbed ends per focus over all simulation time-steps in **(A–C)**. **(H)** Relative frequency of the number of actin polymerization foci. **(I)** Mean lifetime of a focus.

The conversion factor between force imbalance and movement ζ can be expected to have a strong influence on the magnitude of spine shape change per time-step. Judging from the dynamics shown in Figures 7A–C, the area fluctuations also seem to be much faster when increasing ζ. However, this is mostly due to an increase in the amplitude of the fluctuations (Figure 7E) whereas the timescale of the autocorrelation decay remains relatively constant. Note that an increase of ζ enhances spine fluctuation extent, and it also affects the membrane geometrical properties, the membrane force and, hence, the barbed end branching rate. This leads to less barbed ends (Figure 7G) and a reduction of the foci lifetime (Figure 7I). Still, in sum, spines with different values of ζ have similar mean area over time (Figure 7D).

#### 3.3.5. Nucleation Rate γ_*f*_ and Location λ

**Figure 8:**
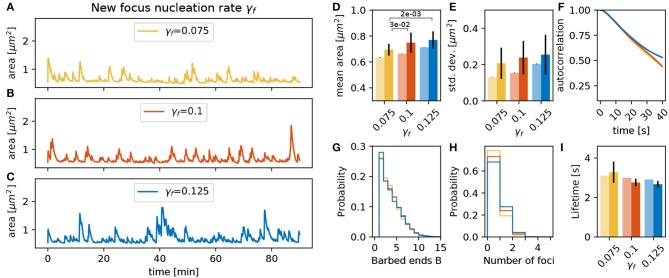
Varying the focus nucleation rate γ_*f*_. **(A–C)** Evolution of the spine head area over time for different values of γ_*f*_: **(A)** γ_*f*_ = 0.075, **(B)** γ_*f*_ = 0.100, and **(C)** γ_*f*_ = 0.125. **(D)** Temporal mean of the spine head area. Light bars correspond to values from single 90 min simulations (standard deviations obtained from 50-fold bootstrap). Full colored bars and errors correspond to the mean and standard deviation over fifteen 15 min simulations. The *p*-value for significant Welch-tests is indicated. **(E)** Standard deviation of the spine head area over time. **(F)** Autocorrelation functions for the area fluctuations in the 90 min simulations. **(G)** Relative frequency of the mean number of barbed ends per focus over all simulation time-steps in **(A–C)**. **(H)** Relative frequency of the number of actin polymerization foci. **(I)** Mean lifetime of a focus.

Additionally to the parameters that influence force generation and translation to movement, the parameters of the nucleation mechanism proposed in section 3.2 can have a strong influence. First, we vary the nucleation rate γ_*f*_ at an intermediate value of the PSD distance scaling parameter λ. As expected, an increase in γ_*f*_ raises the number of actin polymerization foci and the spine area over time (Figure 8H). This leads to a significant increase in the mean area and a trend toward increasing standard deviations (Figures 8D,E). Although these foci have slightly shorter lifetimes (Figure 8I), the decay of the autocorrelation remains at the same timescale (Figure 8F). The main reason for the reduction of foci lifetime is the feedback between the number of barbed ends and the branching rate in Equation (1). If *B* increases then γ_*branch*_ decreases ensuring a limited number of barbed ends at the actin polymerization foci.

**Figure 9:**
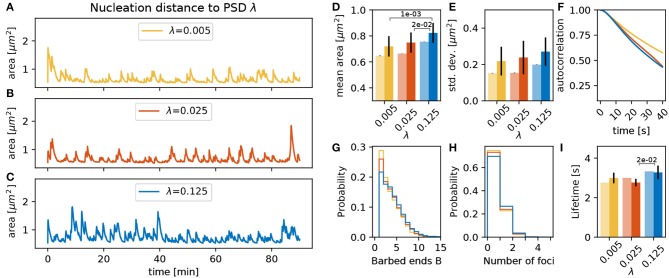
Varying the nucleation distance parameter λ. **(A–C)** Evolution of the spine head area over time for different values of λ: **(A)** λ = 0.005, **(B)** λ = 0.025, and **(C)** λ = 0.125. **(D)** Temporal mean of the spine head area. Light bars correspond to values from single 90 min simulations (standard deviations obtained from 50-fold bootstrap). Full colored bars and errors correspond to the mean and standard deviation over fifteen 15 min simulations. **(E)** Standard deviation of the spine head area over time. The *p*-value for significant Welch-tests is indicated. **(F)** Autocorrelation functions for the area fluctuations in the 90 min simulations. **(G)** Relative frequency of the mean number of barbed ends per focus over all simulation time-steps in **(A–C)**. **(H)** Relative frequency of the number of actin polymerization foci. **(I)** Mean lifetime of a focus.

The location for the polymerization of new foci depends on the distance from the PSD scaled by parameter λ, as stated in section 2.1.3. For larger values of λ, the nucleation points are more likely to be located far from the PSD and the spine mean area is larger due an increase in the lifetime of the actin foci (Figures 9E–I). We speculate that this can be explained by the fact that for small λ all foci nucleate close to the PSD. Hence, all foci push outward the same small fraction of the membrane, which thereby assumes a strong curvature. This, in turn, leads to a strong counteracting force and hence a shorter lifetime of the foci.

In conclusion, we find that geometrical constraints as well as parameters related to actin filament assembly, force generation and focus nucleation have a strong influence on the emerging fluctuation. We summarized the most prominent effects in Table 2.

**Table 2:** Summary table.

**Increase in**	**Mean area**	**Standard deviation**	**Foci lifetime**
PSD-size	⇑	–	–
Branching rate amplitude ϕ	⇑	⇑	⇑
Lateral extent of F-actin σ	⇑	⇑	⇑
Movement speed ζ	–	⇑	⇓
Nucleation rate γ_*f*_	⇑	↑	↓
Nucleation location λ	⇑	↑	–

*Here ↑ (↓) denotes a tendency to increase (decrease) the mean area, standard deviation of the area or foci lifetime whilst ⇑ (⇓) indicates a significant increase (decrease)*.

#### 3.3.6. Influence of Parameter Variation on Spine Area

**Figure 10:**
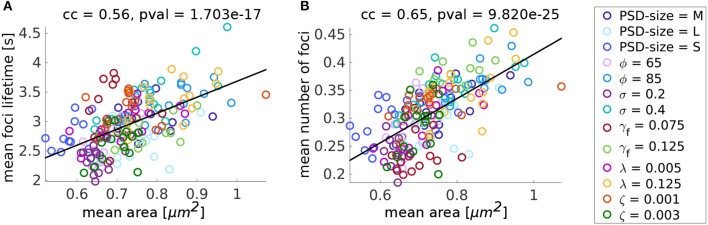
Summary of the parameter variation simulations. **(A)** Mean focus lifetime over mean spine area for different parameter variations. Each dot represents one simulation with a parameter variation corresponding to its color. Standard values of the parameters are reported in Table 1. For every variation of a parameter 15 simulations of 15 min were performed. cc denotes the linear correlation coefficient and pval is the *p*-value for testing the hypothesis of no correlation against the alternative hypothesis of a non-zero correlation, using Pearson's Linear correlation coefficient. **(B)** Same for mean number of foci over mean area.

After evaluating the influence of individual parameters, we investigated whether there are general relations between the evaluated quantities that are preserved over all these variations. To investigate this, we used the fifteen 15 min simulations for each parameter variation and plotted the values of mean area, focus lifetime and mean number of foci for each of these individual simulations against each other. On the one hand, we find that spines with greater mean area over time, have larger mean foci lifetimes (Figure 10A). However, spines with smaller mean area can also have long-lasting foci when the force generated by the membrane is not affecting the branching rate strongly. For example, when the focus nucleation rate γ_*f*_ is high or the movement speed ζ is small. On the other hand, there is a positive correlation between the mean number of actin polymerization foci and spine mean area (Figure 10B), which has also been found in experimental data from Frost et al. (2010). These results imply that the macroscopic spine area fluctuation is heavily relying on the stochastic dynamics of the actin polymerization foci and filament dynamics therein.

### 3.4. Correlation of the Number of Foci and Spine Area Fluctuations

**Figure 11:**
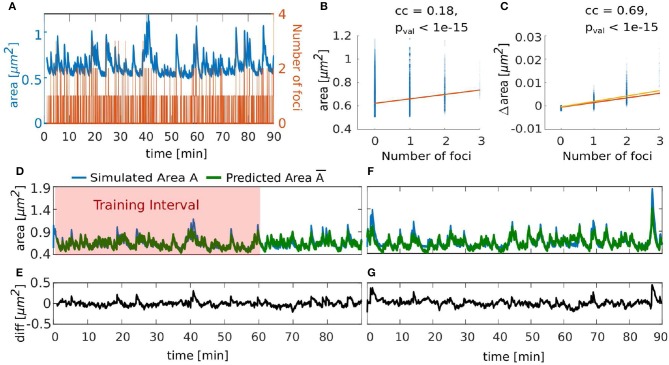
Temporal evolution of spine area can be predicted by number of actin polymerization foci. **(A)** Evolution of number of actin polymerization foci (orange line) and spine area (blue line). **(B)** Spine area over the number of actin polymerization foci for each time-step of the simulation. Color saturation indicates overlay of data points; darker regions contain more data observations. Orange line is a linear fit to the data, cc denotes the linear correlation coefficient and pval is the *p*-value for testing the hypothesis of no correlation against the alternative hypothesis of a non-zero correlation, using Pearson's Linear Correlation Coefficient. **(C)** Same for the difference in spine area over the number of actin polymerization foci. Yellow line is a linear function using the parameters fitted to Equation (8) in **(D)**. **(D)** Simulated area (blue line) and its approximation by estimator $\bar{A}$ (green line, Equation 8) over time. Red shaded zone depicts the training data set for fitting the estimator parameters. **(E)** Difference between simulated and predicted area in **(D)**. **(F)** Simulated area (blue line) corresponding to the plot in Figure 5B and its approximation (green line) over time. **(G)** Difference between the simulated and predicted area in **(F)**.

Although the spine shape is determined by a complex interplay between forces emerging from actin activity and the geometrical properties of the membrane, the above described correlations indicate that there is a strong link between spine area and its polymerization foci. Therefore, we investigated whether the number of polymerization foci at each time-step can be used to predict not only the mean but also the time-course of the spine head size, which is commonly measured in experiments. As the expanding force in our model comes from the actin foci, we first tested whether there is a relationship between number of actin polymerization foci and the spine head area. To quantify this, we tracked the area and the number of foci throughout a 90 min simulation of a spine (Figure 11A) and evaluated the correlation between these quantities. We found a significant correlation, but with a very small correlation coefficient (Figure 11B). When examining the time courses in Figure 11A, we see that when there is no focus the area shrinks to a state close to the resting shape area and a slight increase in area when the number of foci increases. Hence, we investigated the relationship between the actin foci and spine area changes Δarea (Figure 11C) and found that there is indeed a significant correlation with a high correlation coefficient between these quantities. Thus, we constructed a simple model that predicts the area of a spine using only the number of foci at a given time-step. Apart from the area change being proportional to the number of foci, we also included mean retrieval that drives the area back to the area of the Helfrich resting shape *A*_*s*_. In particular, the estimator $\bar{A}$ for the spine area *A* at each time-step *t*_*j*_ is recursively calculated by the following model

(8) $$\begin{array}{l} \bar{A}\left(t_{j}\right)=\bar{A}\left(t_{j-1}\right)-\Phi \left(\bar{A}\left(t_{j-1}\right)-A_{s}\right)+mn_{f}\left(t_{j}\right)+b, \end{array}$$

where the term *mn*_*f*_(*t*_*j*_) + *b* accounts for the change of area that scales linearly with the number of actin polymerization foci *n*_*f*_ at time *t*_*j*_ and Φ represents a decay rate to the resting area *A*_*s*_, which we extracted from our simulations. The model parameters *m, b* and Φ from Equation (8) and the initial area $\bar{A}\left(t_{0}\right)$ were fitted using the fit.m function in MATLAB with the non-linear least square method and the area trace of Figure 11A from min 1 to 60 (Figure 11D, fit results: root mean square error (RMSE) = 0.0562, $\bar{A}\left(t_{0}\right)=0.7507,m=0.002734,b=-0.0004135,\Phi =0.002734$). Hereby, the obtained values for *b* and *m* are close to a linear fit to the relation between the number of foci and the change of the area (orange line in Figure 11C; $\Delta \text{area}=m^{\prime }n_{f}+b^{\prime }$, with *m*′ = 0.00197 and *b*′ = −0.000570). Also, and $\bar{A}\left(t_{0}\right)$ is close to the actual starting value *A*(*t*_0_) = 0.7239. Given that our area estimator is recursive and could accumulate errors over time, $\bar{A}$ performs well, even for a time interval that it was not fitted to (Figure 11D from min 60 to 90, RMSE = 0.0652). Moreover, it performed well when applied to a different simulation with the same parameters (Figure 11F, RMSE = 0.0822). Note that the estimator error increases in periods with large areas (Figures 11E,G), which may be due to the fact that the relation between foci and the change in area may be non-linear (compare Figure 11C). Nevertheless, we deduce that area fluctuations can be predicted very well from the number of actin polymerization foci. Because the fitted model performed well with different simulations, we can conclude that the behavior of the spine area over time is similar regardless of the stochasticity of the model. Thus, already such a relatively simple model gives a good description of the area dynamics. This again underlines a strong link between the microscopic stochastic dynamics at the actin polymerization foci and the macroscopic area fluctuations. Note, however, that this simple model cannot be used as a surrogate for the complete model proposed in this paper, as it relies on knowledge about the number of foci, which is, in turn, only obtainable by stimulating the full non-linear interaction between actin and membrane geometry.

### 3.5. Model Extension to 3D

So far we have only considered spine shapes in 2D, but in order to verify if the dynamics of actin polymerization foci influence spine shape fluctuations in real three-dimensional spines in a similar way, we extended our model to 3D. In this extended model, actin dynamics are preserved but the membrane mesh, all positions and forces are adapted to 3D (see Supplementary Material). Note that the calculation of geometrical properties in 3D is more complex, as more neighboring vertices must be considered. Furthermore, the 3D mesh contains far more vertices than the 2D mesh. Thus, the 3D simulations are computationally expensive and rigorous statistical analysis, as conducted for the 2D model above, is not feasible.

**Figure 12:**
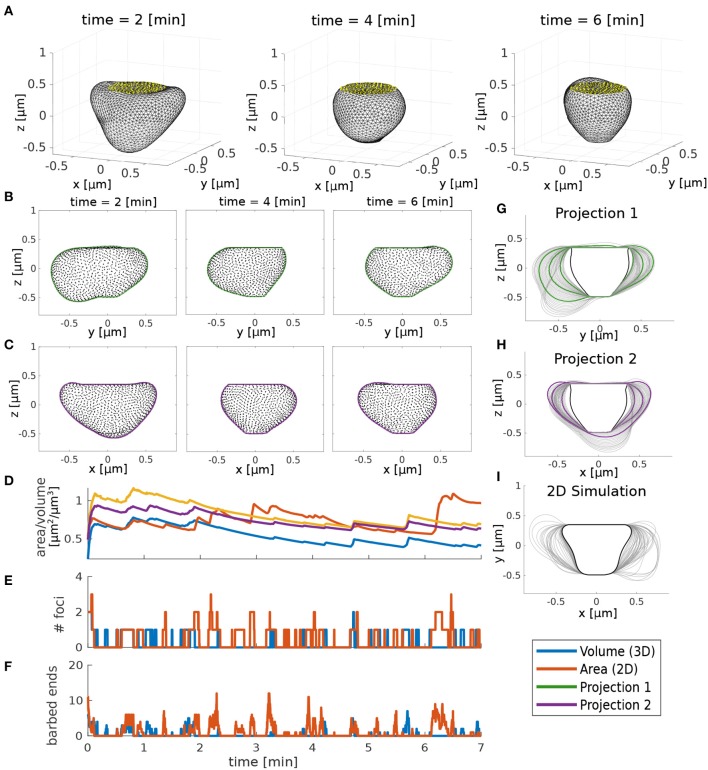
Comparison between 3D and 2D model. **(A)** 3D simulation snapshots at different time points. Yellow dots indicate the PSD. **(B,C)** Projection of the 3D simulation to two 2D views. Black dots are the corresponding 3D vertices and lines are a fitted boundary around these points. **(D)** Time evolution of volume (3D simulation) and area (2D simulation and 2D projections). **(E)** Number of actin foci during the 2D/3D simulation. **(F)** Mean number of barbed ends. **(G,H)** Spine shapes emerging from the 2D projections of the 3D model in time intervals of 10 s. Black lines represent the initial shape and green and purple lines correspond to the snapshots in **(B,C)**, respectively. **(I)** Spine shapes emerging from 2D simulation of the model. Parameters for 3D and 2D simulations are in Table 1 except that in 3D δ_*s*_ = 0.06, ζ = 0.004, and ϕ = 30, and in 2D ζ = 0.0015.

However, we wanted to check whether the qualitative behavior of the 3D and the 2D simulation are comparable. For this, we assumed that the 3D spine shape is observed as in a microscope and projected to a two-dimensional plane. Hereby, we performed projections from multiple sides, in particular to the y-z- and the x-z-plane (Figures 12B–C,G–H, respectively). Deformations in the 3D model where the acting forces are not in the projection plane will appear to be effectively slowed down in 2D projections. To compensate for this, the movement speed of the 2D model has been slowed down by adjusting the movement speed ζ. Moreover, multiple foci in the 3D model may be projected onto the same 2D bump such that it appears as if the foci are more long-lived. To compensate for this, an adjustment in the branching rate amplitude ϕ allows the 2D model to have more barbed ends such that the foci last longer. After adjusting the parameters for these geometrical properties, 3D simulations exhibit qualitatively similar fluctuations to 2D simulations. Hence, we assume that the parameter dependencies and relations between molecular and geometric dynamics discussed above apply similarly to the three-dimensional model.

## 4. Discussion

We proposed a model for dendritic spine shape fluctuations based on actin polymerization foci. We used the model to simulate the dynamics of a single focus as well as the interaction of multiple foci within a spine in 2D and 3D and analyzed the resulting fluctuations of the spine head size. We find that the fluctuations from the projected 3D model are similar to the area fluctuations from the 2D model. Variations of the model parameters revealed that the properties of the molecular processes and mechanics have strong influence on the emergent shape fluctuations. Along these lines, we showed that the changes of the spine head size could be very well-predicted from only knowing how many foci were active over time. Importantly, we showed that the lifetime and hence, the number of foci result from the highly non-linear interactions between actin and membrane forces revealing the importance of embedding actin dynamics on a realistic membrane. Thus, our model provides a platform to study the relation between molecular and morphological properties of the spine.

The proposed model is, to our knowledge, the first to reproduce the rapid asymmetric shape fluctuations observed in experiments. Although the functional role of these fluctuations is an open question, there is the hypothesis that the dynamic actin pool generating the fluctuations is necessary to maintain the spine volume by a dynamic equilibrium (Honkura et al., 2008), and that the dynamic F-actin distribution in the discrete polymerization foci optimizes the spine reaction to plasticity-related events (Frost et al., 2010). In the future, the here presented model can be extended to test this hypothesis.

In our model the asymmetric shape fluctuations result from local imbalances between forces generated by membrane deformation and forces generated by the active actin polymerization foci. Strikingly, these foci have a limited lifetime due to the stochastic nature of the actin filament dynamics. Thereby, the stochasticity of actin dynamics is also transferred onto the spine shape and size, which is evidenced by the fact that the number of active foci can predict the spine area (Figures 10, 11). Our model shows that the focus lifetime is inversely proportional to the force generated by the spine membrane (Figure 2), which is caused by a feedback between this force and the branching rate. This mechanism, thus, couples geometric properties and molecular dynamics, and links the dynamics of multiple foci via the membrane. Moreover, it exhibits that spine size can be maintained by the dynamic actin pool, as proposed by Honkura et al. (2008), while allowing large shape changes seen in Fischer et al. (1998).

Due to the limited lifetime of the actin polymerization foci, we proposed a nucleation that mechanism stochastically generates foci at different locations in the spine. The generation rate and the initial location of these new foci have a great impact on the evolution of spine area over time. For example, an increase of the nucleation rate causes increases of the mean spine area and its standard deviation (Figure 8). Interestingly, foci generated with fast nucleation rate also tend to have a shorter lifetime evidencing a saturating mechanism or self-regulation (compare Statman et al., 2014). Moreover, we used our model to test the influence of the nucleation location of these foci. Experimentally, it has been observed that actin foci are mainly located at the tip of the spine (Honkura et al., 2008; Frost et al., 2010) and the branching protein Arp2/3 is mainly located in a doughnut-shaped zone around the PSD (Rácz and Weinberg, 2008). Such a constraint on the nucleation location of polymerization foci has a strong impact on the shape fluctuations of our model-spines (Figure 9): When foci nucleate closer to the PSD, they tend to last for shorter time intervals such that the mean number of foci is smaller which, in turn, reduces the mean area of the spine. This demonstrates that changes in the polymerization activity can be caused only by differences in geometry without changing any reaction rates.

Furthermore, we observed that, despite the change of shape, the spine area always fluctuates around a mean value, in agreement with experimentally observed spine fluctuations on short timescales (Fischer et al., 1998). This mean value, as well as the magnitude and timescale of the fluctuations are affected by various model parameters. For example, there is a strong influence of the PSD-size on the mean spine area (Figure 4) which is in line with the experimentally observed correlation between these quantities (Boyer et al., 1998; Arellano et al., 2007). Similarly, reducing the branching rate in our model by decreasing ϕ leads to a decrease in the mean and standard deviation of spine area (Figure 5), which is in line with findings that the branching factor Arp2/3 is necessary for spine enlargement and maintenance of spine morphology (Kim et al., 2013). Furthermore, an increase of the movement speed parameter ζ leads to a increase in spine area standard deviation (Figure 7), which has been similarly observed experiments that artificially decreased the density of the extra-cellular matrix in visual cortex (De Vivo et al., 2013). Overall, these results indicate that the mean spine size as well as the magnitude and timescale of spine shape fluctuations are regulated by the properties of the underlying molecular processes (e.g., reaction rates, force generation). Therefore, our model can represent a broad variety of different fluctuation characteristics as observed in experiments through different parameterizations. Moreover, our model agrees with experimental observations that spine size changes, which are not just fluctuations but, instead, affect the mean spine size over longer periods of time or lead to spine loss, result from processes different from actin polymerization (Fischer et al., 1998). Such processes have a longer timescale than that used in this study and may involve the induction of LTP or LTD that can affect the dynamics of actin polymerization. For example, alterations that affect actin-binding proteins change the mean spine size and the spine density (Fortin et al., 2012). Interestingly, this can lead to spine abnormalities that are present in brain-related disorders, such as Alzheimer's disease (Lin and Webb, 2009; Bellot et al., 2014), where memory storage is heavily affected. Thus, it appears that processes that alter the production or function of actin-related proteins, which lead to prominent changes of the spines, can interfere with memory, while spontaneous shape fluctuations presented in this study may aid memory storage by maintaining the spines's typical characteristics.

In conclusion, our model can serve as a basis to investigate the relation between microscopic properties like molecular dynamics, membrane geometry and emerging properties as spine volume fluctuations. As such, it can be extended into various directions: On the one hand, the shape fluctuations may influence the model parameters, such as PSD size, molecule concentrations and reaction rates on longer timescales. Hence, the mean area around which the spine fluctuates as well as other fluctuation characteristics could be continuously adapted giving rise to a slower feedback-loop (compare Yasumatsu et al., 2008 and Statman et al., 2014). On the other hand, so far, the proposed model only considers spines at basal neuronal activity. However, in the future, it can be extended to include induction of activity-dependent plasticity (LTP/LTD) by modeling the changes in actin treadmilling process during plasticity (compare Bennett et al., 2011).

## Data Availability Statement

The code for 2D and 3D simulations is available from GitHub (https://github.com/MayteBQ/Dendritic-Spine-Simulation). Any additional data or code will be made available by the authors, without undue reservation, to any qualified researcher.

## Author Contributions

MB-Q, FW, CT, and MF contributed to the study concept and edited the paper. MB-Q developed the model and performed the simulations. MB-Q and MF analyzed the results and wrote the original draft.

### Conflict of Interest

The authors declare that the research was conducted in the absence of any commercial or financial relationships that could be construed as a potential conflict of interest.
