# Supplementary material for: Modeling the Shape of Synaptic Spines by Their Actin Dynamics
Source: Front Synaptic Neurosci. 2020 Mar 10;12:9. doi: 10.3389/fnsyn.2020.00009 (PMC7078677; doi:10.3389/fnsyn.2020.00009)
Supplement: Supplementary file 1 [file Data_Sheet_1.PDF]

![frontiers]

Supplementary Material

1 MEMBRANE MESH INITIATION

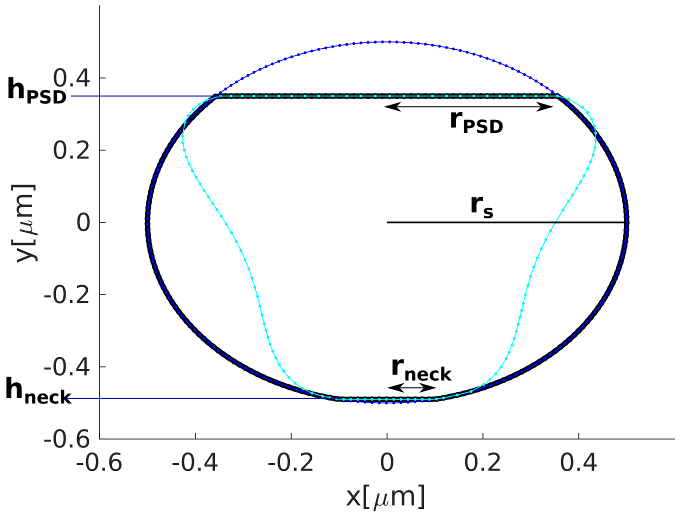

Figure S1. Schematic depiction of the mesh initiation (see Sec. 2.1.1) and resting shape of the modeled dendritic spine. Initially, a circle with radius r_s is drawn (in blue), then the y-coordinate of the vertices (x, y) with $y \geq h_{PSD}$ or $y \leq h_{neck}$ changes to $y = h_{PSD}$ or $y = h_{neck}$, respectively, resulting in a flat circle (in black). Finally, the spine settles to a resting shape (in cyan) when only membrane forces are considered in the simulation.

2 ESTIMATING THE CONCENTRATION OF AVAILABLE ACTIN

Here we derive the steady state concentration of available profilin-ATP-actin a in the spine, that regulates the speed of polymerization, following the model of Bennett et al. (2011) in which the molecular complexes and reactions involved in the recycling of actin are given by

$$\frac{\mathrm{d}a}{\mathrm{d}t} = k_2 p - J_p, \quad \frac{\mathrm{d}p}{\mathrm{d}t} = -k_2 p + k_1 s - k_{-1} p, \quad \frac{\mathrm{d}s}{\mathrm{d}t} = -k_1 s + k_{-1} p + J_d, \tag{S1}$$

where k_2 is the PAD $\rightarrow$ PAT reaction rate, k_1 is the PAD $\rightarrow$ CAD reaction rate and k_{-1} is the CAD $\rightarrow$ PAD reaction rate. J_d is the depolymerization rate and J_p is the polymerization rate. Note that the drift and diffusion of molecules are not modeled explicitly. In the following, all reactions are assumed to be in a steady state such that $J_p = J_d = J$. Furthermore, an increase in profilin leads to an increase in polymerization, hence, $J = ja$. Therefore, at its equilibrium

$$a([P]) = \frac{G}{1 + \frac{j}{k_2} + \frac{j}{k_1([P])}\left(1 + \frac{k_{-1}([P])}{k_2}\right)}. \tag{S2}$$

In Bennett et al. (2011), the values of k_1 and k_{-1} are obtained by assuming that ADP-actin in the association/dissociation reactions of PAD and CAD rapidly approaches its equilibrium, thus

$$k_1([P]) = \frac{k_+^{PD}[P]k_-^C}{k_+^C[S] + k_+^{PD}[P]}, \quad k_{-1}([P]) = \frac{k_+^C[S]k_-^{PD}}{k_+^C[S] + k_+^{PD}[P]}. \tag{S3}$$

For more details see (Bennett et al., 2011), values of rates k_+^{PD}, k_-^{PD}, k_+^C, k_-^C and cofilin concentration $[S]$ are given in Table S1. The value of the fraction of free profilin is given by $[P] = \varphi P_{tot}$, where φ evolves according to

$$\frac{\mathrm{d}\varphi}{\mathrm{d}t} = q_1 - q_2\varphi, \tag{S4}$$

with q_1 and q_2 constant. At its steady state $\varphi = q_1/q_2$, and hence, $[P] = q_1 P_{tot}/q_2$ in Eq. S2. Note however, that the above equations can be used to include LTP by regulating q_1 and q_2 (see Bennett et al. (2011) for details).

Symbol	Definition	Value	Source
k_2 (s^{-1})	PAD $\to$ PAT reaction rate	20	Bennett et al. (2011)
j (s^{-1})	proportionallity constant	5	fitted to match the results in Bennett et al. (2011)
k_+^{PD} ($\mu\mathrm{M}^{-1}\mathrm{s}^{-1}$)	PAD association rate	15	Bennett et al. (2011)
k_-^{PD} (s^{-1})	PAD dissociation rate	10	Bennett et al. (2011)
k_+^C ($\mu\mathrm{M}^{-1}\mathrm{s}^{-1}$)	CAD association rate	150	Bennett et al. (2011)
k_-^C (s^{-1})	CAD dissociation rate	20	Bennett et al. (2011)
$[S]$ (μM)	Total free cofilin	300	Bennett et al. (2011)
G (μM)	concentration of total G-actin	250	Bennett et al. (2011)
P_{tot} (μM)	Total profilin	500	Bennett et al. (2011)
q_1 (s^{-1})	ROCK dissosiation rate, see Eq. (S4)	1.1667×10^{-4}	Bennett et al. (2011)
q_2 (s^{-1})	see Eq. (S4)	0.0033	Bennett et al. (2011)

Table S1. Parameters used to derive avalable actin

3 CALCULATING 2D MEMBRANE FORCES

Following Doubrovinski and Kruse (2011), to calculate the forces acting on the vertices of the polygon approximating the spine head membrane Γ, the free energy associated with Γ

$$\mathcal{E}_{mem} = P\Omega + \tau S + 2\kappa \int_\Gamma (H^2)\mathrm{d}\varpi \tag{S5}$$

is considered. Here, P is the difference between the internal and external pressure, Ω the enclosed area, τ the line tension, S the boundary length, κ the bending modulus, H the mean curvature, and ϖ the arc length of the membrane.

3.1 Area

In 2D, the spine shape can be viewed as a polygonal curve given by n two dimensional vertex with positions $\{\mathbf{x}^1, \mathbf{x}^2, \ldots, \mathbf{x}^n\} = \{(x_1^1, x_2^1), (x_1^2, x_2^2), ..., (x_1^n, x_2^n)\}$. The spine shape is circular, so the ith vertex at position $\mathbf{x}^i$ has neighbours at $\mathbf{x}^{i-1}$ and $\mathbf{x}^{i+1}$, and the last vertex is connected to the first (hence if $i = n$ then $\mathbf{x}^{i-1} = \mathbf{x}^{n-1}$ and $\mathbf{x}^{i+1} = \mathbf{x}^1$, and so on). The area Ω in (S5) can be calculated using the

Gauss-determinant:

$$\Omega = \frac{1}{2}\left(\ldots + \begin{vmatrix} x_1^i & x_1^{i+1} \\ x_2^i & x_2^{i+1} \end{vmatrix} + \begin{vmatrix} x_1^{i+1} & x_1^{i+2} \\ x_2^{i+1} & x_2^{i+2} \end{vmatrix} + \ldots\right), \tag{S6}$$

thus,

$$\Omega = \frac{1}{2}\sum_{i=1}^{n} x_1^i x_2^{i+1} - x_2^i x_1^{i+1}. \tag{S7}$$

3.2 Boundary length

The boundary length S is given by

$$S = \sum_{i=1}^{n} z^i \tag{S8}$$

where z^i, the length of the boundary element corresponding to the vertex i, is defined as

$$z^i = \frac{v^i + v^{i+1}}{2}, \quad i = 1, 2, \ldots, n \tag{S9}$$

with

$$v^i = \| \mathbf{x}^i - \mathbf{x}^{i-1} \| = \sqrt{\left(x_1^i - x_1^{i-1}\right)^2 + \left(x_2^i - x_2^{i-1}\right)^2}. \tag{S10}$$

3.3 Mean curvature

The curvature is given by

$$H = \left\| \frac{\mathrm{d}\mathbf{T}}{\mathrm{d}\varpi} \right\| \tag{S11}$$

where $\mathrm{d}\mathbf{T}$ is the derivative of the unitary tangent vector $\mathbf{T}$ with respect to the arc length ϖ. Assuming that the vertices are close enough, $\mathbf{T}$ can be defined as

$$\mathbf{T}^i = \left(\frac{x_1^i - x_1^{i-1}}{v^i}, \frac{x_2^i - x_2^{i-1}}{v^i}\right), \tag{S12}$$

with v^i as in Eq. S10. Then, using forward finite differences,

$$\frac{\mathrm{d}\mathbf{T}^i}{\mathrm{d}\varpi^i} = \frac{1}{z^i}\left(\frac{x_1^{i+1} - x_1^i}{v^{i+1}} - \frac{x_1^i - x_1^{i-1}}{v^i}, \frac{x_2^{i+1} - x_2^i}{v^{i+1}} - \frac{x_2^i - x_2^{i-1}}{v^i}\right), \tag{S13}$$

and therefore

$$(H^i)^2 = \frac{1}{(z^i)^2}\left(\left(\frac{x_1^{i+1} - x_1^i}{v^{i+1}} - \frac{x_1^i - x_1^{i-1}}{v^i}\right)^2 + \left(\frac{x_2^{i+1} - x_2^i}{v^{i+1}} - \frac{x_2^i - x_2^{i-1}}{v^i}\right)^2\right) =: \frac{g^i}{(z^i)^2}. \tag{S14}$$

3.4 Membrane-force

In the 2D case, (S5) can be rewritten as

$$\mathbf{F}^i_{mem} = -P\frac{\partial \Omega}{\partial \mathbf{x}^i} - \tau\frac{\partial S}{\partial \mathbf{x}^i} - 2\kappa\int_\Gamma \frac{\partial H^2}{\partial \mathbf{x}^i}\mathrm{d}\varpi, \tag{S15}$$

where

$$\frac{\partial \Omega}{\partial x^i_1} = \frac{1}{2}\left(x_2^{i+1} - x_2^{i-1}\right), \quad \frac{\partial \Omega}{\partial x^i_2} = \frac{1}{2}\left(-x_1^{i+1} + x_1^{i-1}\right), \tag{S16}$$

and

$$\begin{aligned}\frac{\partial S}{\partial x^i_{1,2}} &= \frac{\partial}{\partial x^i_{1,2}}\left(z^{i-1} + z^i + z^{i+1}\right) = \frac{\partial}{\partial x^i_{1,2}}\left(\frac{v^{i-1}+v^i}{2} + \frac{v^i + v^{i+1}}{2} + \frac{v^{i+1}+v^{i+2}}{2}\right) \\ &= \frac{x^i_{1,2} - x^{i-1}_{1,2}}{v^i} + \frac{x^i_{1,2} - x^{i+1}_{1,2}}{v^{i+1}}.\end{aligned}$$

Here $x^i_{1,2}$ means that the equation hold for both the first or second coordinate position.

For the curvature term, Doubrovinski and Kruse (2011) assumed $\mathrm{d}\varpi \simeq z$, hence

$$\begin{aligned}\int_\Gamma \frac{\partial H^2}{\partial \mathbf{x}^i}\mathrm{d}\varpi &\simeq \frac{\partial}{\partial x^i_{1,2}}\sum_j \frac{1}{z^j}g^j = \sum_j \frac{1}{z^j}\frac{\partial g^j}{\partial x^i_{1,2}} - \frac{g^j}{(z^j)^2}\frac{\partial z^j}{\partial x^i_{1,2}} \\ &= \frac{1}{z^{i-1}}\frac{\partial g^{i-1}}{\partial x^i_{1,2}} - \frac{g_{i-1}}{(z^{i-1})^2}\frac{\partial z^{i-1}}{\partial x^i_{1,2}} + \frac{1}{z^i}\frac{\partial g^i}{\partial x^i_{1,2}} - \frac{g^i}{(z^i)^2}\frac{\partial z^i}{\partial x^i_{1,2}} + \frac{1}{z^{i+1}}\frac{\partial g^{i+1}}{\partial x^i_{1,2}} - \frac{g^{i+1}}{(z^{i+1})^2}\frac{\partial z^{i+1}}{\partial x^i_{1,2}},\end{aligned}$$

where

$$\begin{aligned}\frac{\partial z^{i-1}}{\partial x^i_{1,2}} &= \frac{1}{2}\left(\frac{x^i_{1,2} - x^{i-1}_{1,2}}{v^i}\right), \\ \frac{\partial z^i}{\partial x^i_{1,2}} &= \frac{1}{2}\left(\frac{x^i_{1,2} - x^{i-1}_{1,2}}{v^i} - \frac{x^{i+1}_{1,2} - x^i_{1,2}}{v^{i+1}}\right), \\ \frac{\partial z^{i+1}}{\partial x^i_{1,2}} &= -\frac{1}{2}\left(\frac{x^{i+1}_{1,2} - x^i_{1,2}}{v^{i+1}}\right),\end{aligned}$$

and

$$
\begin{aligned}
\frac{\partial g^{i-1}}{\partial x_1^i} &= 2\left(\frac{x_1^i - x_1^{i-1}}{v^i} - \frac{x_1^{i-1} - x_1^{i-2}}{v^{i-1}}\right)\left(\frac{1}{v^i} - \frac{\left(x_1^i - x_1^{i-1}\right)^2}{(v^i)^3}\right) \\
&+ 2\left(\frac{x_2^i - x_2^{i-1}}{v^i} - \frac{x_2^{i-1} - x_2^{i-2}}{v^{i-1}}\right)\left(-\frac{\left(x_2^i - x_2^{i-1}\right)\left(x_1^i - x_1^{i-1}\right)}{(v^i)^3}\right), \\
\frac{\partial g_{i+1}}{\partial x_1^i} &= 2\left(\frac{x_1^{i+2} - x_1^{i+1}}{v^{i+2}} - \frac{x_1^{i+1} - x_1^{i}}{v^{i+1}}\right)\left(\frac{1}{v^{i+1}} - \frac{\left(x_1^{i+1} - x_1^{i}\right)^2}{(v^{i+1})^3}\right) \\
&+ 2\left(\frac{x_2^{i+2} - x_2^{i+1}}{v^{i+2}} - \frac{x_2^{i+1} - x_2^{i}}{v^{i+1}}\right)\left(-\frac{\left(x_2^{i+1} - x_2^{i}\right)\left(x_1^{i+1} - x_1^{i}\right)}{(v^{i+1})^3}\right), \\
\frac{\partial g^{i}}{\partial x_1^i} &= 2\left(\frac{x_1^{i+1} - x_1^{i}}{v^{i+1}} - \frac{x_1^{i} - x_1^{i-1}}{v^{i}}\right)\left(-\frac{1}{v^{i+1}} + \frac{\left(x_1^{i+1} - x_1^{i}\right)^2}{(v^{i+1})^3} - \frac{1}{v^i} + \frac{\left(x_1^{i} - x_1^{i-1}\right)^2}{(v^{i})^3}\right) \\
&+ 2\left(\frac{x_2^{i+1} - x_2^{i}}{v^{i+1}} - \frac{x_2^{i} - x_2^{i-1}}{v^{i}}\right)\left(\frac{\left(x_2^{i+1} - x_2^{i}\right)\left(x_1^{i+1} - x_1^{i}\right)}{(v^{i+1})^3} + \frac{\left(x_2^{i} - x_2^{i-1}\right)\left(x_1^{i} - x_1^{i-1}\right)}{(v^{i})^3}\right)
\end{aligned}
$$

(for the derivatives with respect to x_2^j the subscript indices 1 and 2 are swapped). Note that all term involved in the force calculation at $\mathbf{x}^i$ only include the coordinates of its neighbors $\mathbf{x}^{i-1}$, $\mathbf{x}^{i+1}$ and $\mathbf{x}^{i+2}$.

4 CALCULATING 3D MEMBRANE FORCES

The Helfrich free energy (Guckenberger et al., 2016) is given by

$$\mathcal{E}_{mem} = P \oint \mathrm{d}V + \tau \oint \mathrm{d}A + \frac{\kappa}{2} \oint \mathrm{d}A(2H^2), \tag{S17}$$

where P is the difference between the internal and external pressure, τ the surface tension, and κ the bending modulus. Here V, A and H represent volume, surface area and mean curvature, respectively.

In the 3D model, the discretized spine head membrane is a mesh containing n_v vertices $\mathbf{x}^i = \left(x_1^i, x_2^i, x_3^i\right) \in \mathbb{R}^3$, $i = 1, 2, \ldots, n_v$. The mesh is formed by a set of n_t triangles. The relation between vertices and triangles is given by a triangulation matrix $\mathcal{T} \in \mathbb{R}^{n_t \times 3}$, which contains the index of the vertices forming a triangle I in every row. For the calculations, we assume that a triangle is formed by the vertices at positions $\mathbf{x}^i$, $\mathbf{x}^j$ and $\mathbf{x}^{j-1}$ (see Fig. S2) and its corresponding entry in the triangulation matrix is $\mathcal{T}(I) = (i, j-1, j)$. Note that a triangulation can contain more than one triangle with first entry in the triangulation matrix corresponding to vertex at position $\mathbf{x}^i$ but forming different triangles (different values in the second and third position of the triangulation matrix). Hereinafter, calculations of volume and surface area are based on the mesh triangulation whilst calculations of the curvature are based on the set of vertices.

4.1 Volume

Following Krüger (2012), the first terms in the right-hand-side of (S17) can be rewritten as

$$P \oint \mathrm{d}V \approx P \sum_{I=1}^{n_t} V^I, \quad V^I = \frac{1}{6} \left(\mathbf{x}^{j-1} \times \mathbf{x}^j\right) \cdot \mathbf{x}^i, \tag{S18}$$

where $\times$ represents the cross product and $\cdot$ the scalar product.

4.2 Surface area

Similarly, Krüger (2012) rewrites the second term as

$$\tau \oint \mathrm{d}A \approx \tau \sum_{I=1}^{n_t} A^I, \quad A^I = \frac{1}{2} \left|\mathbf{N}^i\right|, \tag{S19}$$

where $\mathbf{N}^i$ is the outer normal vector of the membrane surface at position $\mathbf{x}^i$ given by

$$\mathbf{N}^i = \left(\mathbf{x}^{j-1} - \mathbf{x}^i\right) \times \left(\mathbf{x}^j - \mathbf{x}^i\right), \tag{S20}$$

and $|\mathbf{x}| = \sqrt{x_1^2 + x_2^2 + x_3^2}$ is the euclidean norm.

4.3 Mean curvature

For the curvature term in (S17) we approximate the integral by summing over the vertices instead of triangles and use the following expression for H (Xu, 2004; Guckenberger et al., 2016; Gompper and Kroll, 1996; Guckenberger and Gekle, 2017):

$$H = \sum_{i=1}^{n_v} H^i, \quad H^i = H(\mathbf{x}^i) = \frac{1}{2} \triangle_{\mathbf{s}} \mathbf{x}^i \cdot \mathbf{n}^i, \quad \mathbf{n}^i = \frac{\mathbf{N}^i}{|\mathbf{N}^i|}, \tag{S21}$$

where $\triangle_{\mathbf{s}}$ denotes the Laplace-Beltrami operator which can be approximated (Gompper and Kroll, 1996) as

$$\triangle_s \, x_l^i \approx \frac{\sum_{j(i)} (\cot\theta_1^{ij} + \cot\theta_2^{ij})(x_l^i - x_l^j)}{2A_V^i}, \quad i = 1, 2, \dots n_v, \quad l = 1, 2, 3, \tag{S22}$$

where the sum runs over the neighbours $j(i)$ of $\mathbf{x}^i$. Ideally, the vertex at position $\mathbf{x}^i$ has 6 neighbours at positions $\mathbf{x}^j$s, where $\mathbf{x}^{j-1}$ ($\mathbf{x}^{j+1}$) denotes the next neighboring[1] vertex of $\mathbf{x}^i$ to the left (right) side of $\mathbf{x}^j$. A_V^i is the Voronoi area corresponding to vertex $\mathbf{x}^i$ and θ_1^{ij} and θ_2^{ij} are the angles opposite to the edge between $\mathbf{x}^i$ and $\mathbf{x}^j$ and in the triangles containing $\mathbf{x}^{j-1}$ and $\mathbf{x}^{j+1}$, respectively (see Figure S2). Their

[1] This notation is independent of the triangulation.

cotangents are given by

$$\cot\theta_1^{ij} = \frac{\cos\theta_1^{ij}}{\sqrt{1-\cos^2\theta_1^{ij}}}, \quad \cos\theta_1^{ij} = \frac{\left(\mathbf{x}^i - \mathbf{x}^{j-1}\right)\cdot\left(\mathbf{x}^j - \mathbf{x}^{j-1}\right)}{l_{i,j-1}l_{j,j-1}}, \tag{S23}$$

$$\cot\theta_2^{ij} = \frac{\cos\theta_2^{ij}}{\sqrt{1-\cos^2\theta_2^{ij}}}, \quad \cos\theta_2^{ij} = \frac{\left(\mathbf{x}^i - \mathbf{x}^{j+1}\right)\cdot\left(\mathbf{x}^j - \mathbf{x}^{j+1}\right)}{l_{i,j+1}l_{j,j+1}}, \tag{S24}$$

$$\text{with} \quad l_{i,j} = \left|\mathbf{x}^i - \mathbf{x}^j\right|. \tag{S25}$$

The Voronoi area A_V^i corresponding to node $\mathbf{x}^i$ is defined by

$$A_V^i = \frac{1}{8}\sum_{j(i)}(\cot\theta_1^{ij} + \cot\theta_2^{ij})\left|\mathbf{x}^i - \mathbf{x}^j\right|^2, \quad i = 1,\ldots,n_v. \tag{S26}$$

Hence, the last right-hand-side term in (S17) can be rewritten as

$$\frac{\kappa}{2}\oint(2H^2)\mathrm{d}A \approx \frac{\kappa}{2}\sum_i(2H^i)^2A_V^i = 2\kappa\sum_i\mathcal{H}_b^i \text{ with } \mathcal{H}_b^i = (H^i)^2A_V^i. \tag{S27}$$

4.4 Membrane force

The force $\mathbf{F}_{mem}^i$ on vertex i located at $\mathbf{x}^i$ of the polyhedron approximating the spine head membrane is given by

$$\mathbf{F}_{mem}^i = -\frac{\partial\mathcal{E}_{mem}^i}{\partial\mathbf{x}^i} = -\left(\frac{\partial\mathcal{E}_{mem}^i}{\partial x_1^i}, \frac{\partial\mathcal{E}_{mem}^i}{\partial x_2^i}, \frac{\partial\mathcal{E}_{mem}^i}{\partial x_3^i}\right). \tag{S28}$$

As stated above the volume and surface area terms are calculated using the triangulation $\mathcal{T}$. Therefore, for the first term of $\mathcal{E}_{mem}$ in Eq. (S17),

$$\frac{\partial}{\partial\mathbf{x}^i}\oint\mathrm{d}V \approx \frac{\partial}{\partial\mathbf{x}^i}\sum_I V^I, \tag{S29}$$

only triangles containing $\mathbf{x}^i$ are taken into account. If $\mathbf{x}^i$ is on the first column of $\mathcal{T}$, then its contribution is $(\mathbf{x}^{j-1}\times\mathbf{x}^j)/6$, on the second column $(\mathbf{x}^j\times\mathbf{x}^i)/6$, and on the third column $(\mathbf{x}^i\times\mathbf{x}^{j-1})/6$. Because one vertex form part of different triangles, these contributions are summed accordingly.

Similarly, for the derivative of the second term of $\mathcal{E}_{mem}$ in Eq. (S17) only the triangles containing $\mathbf{x}^i$ contribute to the force at that point. The contribution depends on the position within the triangulation matrix $\mathcal{T}$. Assuming that the vertex $\mathbf{x}^i$ is in the first column of the triangulation matrix, then it contributes $\left(\mathbf{n}^i\times(\mathbf{x}^j - \mathbf{x}^{j-1})\right)/2$ because the derivative of the surface area at x_k^i is given by

$$\frac{\partial A^I}{\partial x_k^i} = \frac{1}{2}\mathbf{n}^i\cdot\frac{\partial\mathbf{N}^i}{\partial x_k^i}, \quad \frac{\partial\mathbf{N}^i}{\partial x_k^i} = (\mathbf{x}^j - \mathbf{x}^{j-1})\times\hat{\mathbf{e}}_k, \quad k = 1,2,3, \tag{S30}$$

where $\hat{\mathrm{e}}_k$ is the kth unit vector. Likewise, if $\mathbf{x}^i$ is on the second column of the triangulation matrix, then its contribution is $\left(\mathbf{n}^i \times (\mathbf{x}^i - \mathbf{x}^j)\right)/2$, and on the third $\left(\mathbf{n}^i \times (\mathbf{x}^{j-1} - \mathbf{x}^i)\right)/2$. Contributions of different triangles containing $\mathbf{x}^i$ are summed.

Finally, for the derivative of the third term in Eq. (S17) note that $\mathcal{H}_b^i$ in Eq. (S27) is a function of $\mathbf{x}^i$ and its neighbours. Hence, a vertex k have position $\mathbf{x}^i$ when calculating $\mathcal{H}_b^i$, position $\mathbf{x}^j$ when calculating $\mathcal{H}_b^j$, and so on. In the following, we derive the contribution to the derivative $\mathcal{H}_b^i$ for all the points involved in its calculation, thus

$$\frac{\partial \mathcal{H}_b^i}{\partial \mathbf{x}^m} = 2H^i \frac{\partial H^i}{\partial \mathbf{x}^m} A_V^i + (H^i)^2 \frac{\partial A_V^i}{\partial \mathbf{x}^m}, \quad m \in \{i, j, j-1, j+1\}, \tag{S31}$$

where

$$\frac{\partial H^i}{\partial \mathbf{x}^m} = \frac{1}{2}\left(\frac{\partial \triangle_{\mathbf{s}} \mathbf{x}^i}{\partial \mathbf{x}^m} \cdot \mathbf{n}^i + \triangle_{\mathbf{s}} \mathbf{x}^i \cdot \frac{\partial \mathbf{n}^i}{\partial \mathbf{x}^m}\right), \tag{S32}$$

with

$$\begin{aligned} \frac{\partial \triangle_s x_l^i}{\partial x_k^m} &= \frac{1}{2}\sum_{j(i)} \frac{\left(\frac{\partial \cot \theta_1^{ij}}{\partial x_k^m} + \frac{\partial \cot \theta_2^{ij}}{\partial x_k^m}\right)\left(x_l^i - x_l^j\right)}{A_V^i} + \frac{\left(\cot \theta_1^{ij} + \cot \theta_2^{ij}\right)\frac{\partial\left(x_l^i - x_l^j\right)}{\partial x_k^m}}{A_V^i} \\ &- \frac{\left(\cot \theta_1^{ij} + \cot \theta_2^{ij}\right)\left(x_l^i - x_l^j\right)\frac{\partial A_V^i}{\partial x_k^m}}{\left(A_V^i\right)^2}. \end{aligned} \tag{S33}$$

In general,

$$\frac{\partial \cot \theta}{\partial \mathbf{x}^m} = \frac{1}{(1 - \cos^2 \theta)^{3/2}} \frac{\partial \cos \theta}{\partial \mathbf{x}^m}. \tag{S34}$$

Here,

$$\begin{aligned} \frac{\partial \cos \theta_1^{ij}}{\partial \mathbf{x}^i} &= \frac{\mathbf{x}^j - \mathbf{x}^{j-1}}{l_{i,j-1} l_{j,j-1}} - \frac{\cos \theta_1^{ij}\left(\mathbf{x}^i - \mathbf{x}^{j-1}\right)}{l_{i,j-1}^2}, \quad \frac{\partial \cos \theta_2^{ij}}{\partial \mathbf{x}^i} = \frac{\mathbf{x}^j - \mathbf{x}^{j+1}}{l_{i,j+1} l_{j,j+1}} - \frac{\cos \theta_2^{ij}\left(\mathbf{x}^i - \mathbf{x}^{j+1}\right)}{l_{i,j+1}^2}, \\ \frac{\partial \cos \theta_1^{ij}}{\partial \mathbf{x}^{j-1}} &= \frac{2\mathbf{x}^{j-1} - \mathbf{x}^j - \mathbf{x}^i}{l_{i,j-1} l_{j,j-1}} + \cos \theta_1^{ij}\left(\frac{\mathbf{x}^i - \mathbf{x}^{j-1}}{l_{i,j-1}^2} + \frac{\mathbf{x}^j - \mathbf{x}^{j-1}}{l_{j,j-1}^2}\right), \quad \frac{\partial \cos \theta_2^{ij}}{\partial \mathbf{x}^{j-1}} = \mathbf{0}, \\ \frac{\partial \cos \theta_1^{ij}}{\partial \mathbf{x}^j} &= \frac{\mathbf{x}^i - \mathbf{x}^{j-1}}{l_{i,j-1} l_{j,j-1}} - \frac{\cos \theta_1^{ij}\left(\mathbf{x}^j - \mathbf{x}^{j-1}\right)}{l_{j,j-1}^2}, \quad \frac{\partial \cos \theta_2^{ij}}{\partial \mathbf{x}^j} = \frac{\mathbf{x}^i - \mathbf{x}^{j+1}}{l_{i,j+1} l_{j,j+1}} - \frac{\cos \theta_2^{ij}\left(\mathbf{x}^j - \mathbf{x}^{j+1}\right)}{l_{j,j+1}^2}, \\ \frac{\partial \cos \theta_1^{ij}}{\partial \mathbf{x}^{j+1}} &= \mathbf{0}, \quad \frac{\partial \cos \theta_2^{ij}}{\partial \mathbf{x}^{j+1}} = \frac{2\mathbf{x}^{j+1} - \mathbf{x}^j - \mathbf{x}^i}{l_{i,j+1} l_{j,j+1}} + \cos \theta_2^{ij}\left(\frac{\mathbf{x}^i - \mathbf{x}^{j+1}}{l_{i,j+1}^2} + \frac{\mathbf{x}^j - \mathbf{x}^{j+1}}{l_{j,j+1}^2}\right). \end{aligned}$$

Now,

$$\frac{\partial A_V^i}{\partial \mathbf{x}^m} = \frac{1}{8}\sum_{j(i)}\left(\left(\frac{\partial \cot \theta_1^{ij}}{\partial \mathbf{x}^m} + \frac{\partial \cot \theta_2^{ij}}{\partial \mathbf{x}^m}\right) l_{i,j}^2 + \left(\cot \theta_1^{ij} + \cot \theta_2^{ij}\right)\frac{\partial l_{i,j}^2}{\partial \mathbf{x}^m}\right), \quad m \in \{i, j, j-1, j+1\}, \tag{S35}$$

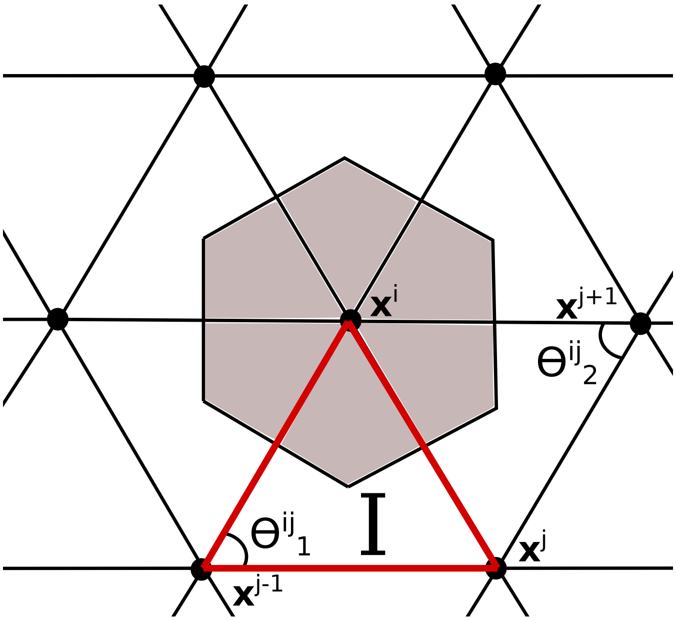

Figure S2. Neighbors of node $\mathbf{x}^i$. Here vertices $\mathbf{x}^i$, $\mathbf{x}^j$ and $\mathbf{x}^{j-1}$ form the triangle I (in red). Gray region: Voronoi Area A^i_V

where

$$\frac{\partial l^2_{i,j}}{\partial \mathbf{x}^i} = 2\left(\mathbf{x}^i - \mathbf{x}^j\right), \quad \frac{\partial l^2_{i,j}}{\partial \mathbf{x}^j} = -2\left(\mathbf{x}^i - \mathbf{x}^j\right), \quad \frac{\partial l^2_{i,j}}{\partial \mathbf{x}^{j-1}} = \frac{\partial l^2_{i,j}}{\partial \mathbf{x}^{j+1}} = \mathbf{0}. \tag{S36}$$

The derivative of the normalized normal vector is given by

$$\frac{\partial \mathbf{n}^i}{\partial \mathbf{x}^m} = \frac{1}{|\mathbf{N}^i|}\left(\frac{\partial \mathbf{N}^i}{\partial \mathbf{x}^m} - \mathbf{n}^i\left(\mathbf{n}^i \cdot \frac{\partial \mathbf{N}^i}{\partial \mathbf{x}^m}\right)\right), \quad m \in \{i, j-1, j, j+1\} \tag{S37}$$

hence

$$\triangle_s \mathbf{x}^i \cdot \frac{\partial \mathbf{n}^i}{\partial \mathbf{x}^m} = \frac{1}{|\mathbf{N}^i|}\left(\triangle_s \mathbf{x}^i - \left(\triangle_s \mathbf{x}^i \cdot \mathbf{n}^i\right)\mathbf{n}^i\right) \cdot \frac{\partial \mathbf{N}^i}{\partial \mathbf{x}^m}. \tag{S38}$$

The expressions for each $m \in \{i, j-1, j, j+1\}$ are

$$\triangle_s \mathbf{x}^i \cdot \frac{\partial \mathbf{n}^i}{\partial \mathbf{x}^i} = \frac{1}{|\mathbf{N}^i|}\left(\triangle_s \mathbf{x}^i - \left(\triangle_s \mathbf{x}^i \cdot \mathbf{n}^i\right)\mathbf{n}^i\right) \times \left(\mathbf{x}^j - \mathbf{x}^{j-1}\right), \tag{S39}$$

$$\triangle_s \mathbf{x}^i \cdot \frac{\partial \mathbf{n}^i}{\partial \mathbf{x}^j} = \frac{1}{|\mathbf{N}^i|}\left(\triangle_s \mathbf{x}^i - \left(\triangle_s \mathbf{x}^i \cdot \mathbf{n}^i\right)\mathbf{n}^i\right) \times \left(\mathbf{x}^{j-1} - \mathbf{x}^i\right), \tag{S40}$$

$$\triangle_s \mathbf{x}^i \cdot \frac{\partial \mathbf{n}^i}{\partial \mathbf{x}^{j-1}} = \frac{1}{|\mathbf{N}^i|}\left(\triangle_s \mathbf{x}^i - \left(\triangle_s \mathbf{x}^i \cdot \mathbf{n}^i\right)\mathbf{n}^i\right) \times \left(\mathbf{x}^i - \mathbf{x}^j\right). \tag{S41}$$

Note that a vertex have many contributions depending on the role it takes in the curvature calculation (main vertex i or neighbouring vertex j,$j-1$,$j+1$), thus we sum these contributions.

REFERENCES

Bennett, M. R., Farnell, L., and Gibson, W. G. (2011). A model of nmda receptor control of f-actin treadmilling in synaptic spines and their growth. *Bulletin of mathematical biology* 73, 2109–2131

Doubrovinski, K. and Kruse, K. (2011). Cell motility resulting from spontaneous polymerization waves. *Physical Review Letters* 107, 258103

Gompper, G. and Kroll, D. (1996). Random surface discretizations and the renormalization of the bending rigidity. *Journal de Physique I* 6, 1305–1320

Guckenberger, A. and Gekle, S. (2017). Theory and algorithms to compute helfrich bending forces: A review. *Journal of Physics: Condensed Matter* 29, 203001

Guckenberger, A., Schraml, M. P., Chen, P. G., Leonetti, M., and Gekle, S. (2016). On the bending algorithms for soft objects in flows. *Computer Physics Communications* 207, 1–23

Krüger, H. (2012). *Computer simulation study of collective phenomena in dense suspensions of red blood cells under shear* (Springer Science & Business Media)

Xu, G. (2004). Discrete laplace–beltrami operators and their convergence. *Computer aided geometric design* 21, 767–784
